# Glucuronidation Pathways of 5- and 7-Hydroxypropranolol: Determination of Glucuronide Structures and Enzyme Selectivity

**DOI:** 10.3390/molecules28237783

**Published:** 2023-11-26

**Authors:** Fan Yang, Maxi Wenzel, Matthias Bureik, Maria Kristina Parr

**Affiliations:** 1 Pharmaceutical and Medicinal Chemistry (Pharmaceutical Analyses), Institute of Pharmacy, Freie Universität Berlin, 14195 Berlin, Germany; yangfano129@zedat.fu-berlin.de (F.Y.); maxi.wenzel@fu-berlin.de (M.W.); 2 School of Pharmaceutical Science and Technology, Tianjin University, Tianjin 300072, China; matthias@tju.edu.cn

**Keywords:** UGTs, hydroxypropranolol, glucuronidation, enzyme bags, drug metabolism, structural investigation

## Abstract

Propranolol, a non-selective beta-blocker medication, has been utilized in the treatment of cardiovascular diseases for several decades. Its hydroxynaphthyl metabolites have been recognized to possess varying degrees of beta-blocker activity due to the unaltered side-chain. This study achieved the successful separation and identification of diastereomeric glucuronic metabolites derived from 4-, 5-, and 7-hydroxypropranolol (4-OHP, 5-OHP, and 7-OHP) in human urine. Subsequently, reaction phenotyping of 5- and 7-hydroxypropranolol by different uridine 5’-diphospho-glucuronosyltransferases (UGTs) was carried out, with a comparison to the glucuronidation of 4-hydroxypropranolol (4-OHP). Among the 19 UGT enzymes examined, UGT1A1, UGT1A3, UGT1A7, UGT1A8, UGT1A9, UGT1A10, UGT2A1, and UGT2A2 were found to be involved in the glucuronidation of 5-OHP. Furthermore, UGT1A6 exhibited glucuronidation activity towards 7-OHP, along with the aforementioned eight UGTs. Results obtained by glucuronidation of corresponding methoxypropranolols and MS/MS analysis of 1,2-dimethylimidazole-4-sulfonyl (DMIS) derivatives of hydroxypropranolol glucuronides suggest that both the aromatic and aliphatic hydroxy groups of the hydroxypropranolols may be glucuronidated in vitro. However, the analysis of human urine samples collected after the administration of propranolol leads us to conclude that aromatic-linked glucuronidation is the preferred pathway under physiological conditions.

## 1. Introduction

Uridin-5′-diphospho-glucuronosyltransferases (UGTs) are a large family of human phase II drug metabolizing enzymes that play a critical role in eliminating many endogenous and exogenous compounds [1]. Together with the major phase I metabolism enzymes, the cytochrome P450s (CYPs), they account for approximately 90% of the metabolism of prescription drugs [2]. Typically, UGTs catalyze glucuronidation reactions by adding a glucuronic acid moiety from the cofactor uridine diphosphate glucuronic acid (UDPGA) to a functional group (usually a hydroxy group) of the target drugs or their phase I metabolite generated by CYPs [3,4]. The resulting glucuronides are more hydrophilic and therefore easier to be excreted via urine. There are 22 human UGTs, with 19 of them belonging to the UGT1 and UGT2 families. These are the primary drug metabolism enzymes [5]. The remaining 3 UGTs are believed to be involved in the homeostasis of endogenous substances [6,7,8].

Propranolol was developed as a non-selective beta-receptor antagonist back in the 1960s [9]. Since then, propranolol has been used to treat a range of cardiovascular diseases [10,11]. At the same time, the anxiolytic effect of propranolol attracted researchers’ attention, and the use of this effect was studied in a range of conditions, such as in the treatment of high trait anxiety and the improvement of the performance of a doctor during eye surgery [12,13,14]. In recent years, propranolol has regained the attention of researchers because of its good therapeutic performance in the treatment of infantile hemangioma, and rare diseases such as hereditary hemorrhagic telangiectasia and cerebral cavernous malformations [15,16,17]. Therefore, it is important and relevant to study the metabolic profile of propranolol in humans. In previous studies, propranolol was reported to undergo three main metabolic pathways. These are naphthyl hydroxylation, *N*-desisopropylation, and side-chain glucuronidation [18,19]. CYP2D6 was found to be the main CYP isoform that catalyzes the hydroxylation of the naphthyl group, with 4-hydroxypropranolol (4-OHP) being the most abundant metabolite, followed by its 5- and 7-hydroxy isomers (5-OHP and 7-OHP) [20,21,22]. Previous studies have highlighted the significance of the side-chain for the pharmacological activity of the drug [23]. Therefore, it is not surprising that the naphthylhydroxylated metabolites of propranolol, including 4-, 5-, and 7-OHP, still possess certain beta-blocking effects due to their unaltered side-chain. Indeed, it has been observed that 4-OHP and 5-OHP both have beta-blocking activity, with 4-OHP even being equipotent to the parental drug itself [24,25,26,27]. The pharmacological activity of 7-OHP is still unknown. In our previous research, we identified UGT1A7, UGT1A8, UGT1A9, and UGT2A1 as responsible UGT isoforms for the glucuronidation of 4-OHP [28]. However, the reaction phenotyping of 5- and 7-OHP remains unclear and requires additional investigation.

It is obvious that the structural identification and assignment of the potential conjugation sites of glucuronides are important aspects in the study of pharmacology and toxicology. The famous example of morphine-6-glucuronide and morphine-3-glucuronide shows that different glucuronic regio-isomers may have vastly different properties [29]. In the case of propranolol, the CYP-catalyzed phase I reaction adds an aromatic hydroxy group, which raises questions about the regioselectivity of glucuronidation on hydroxypropranolols.

During the 1980s, researchers investigated the regioselectivity of 4-OHP glucuronidation using GC-MS/MS after derivatization with diazomethane [30]. The resulting 4-hydroxypropranolol glucuronides (4-OHPGs) obtained from both in vivo and in vitro samples were found to be linked to the aromatic hydroxy group. Subsequently, Salomonsson et al. discovered that both aromatic-linked and aliphatic-linked 4-OHPGs were produced by analyzing in vitro samples derivatized with 1,2-dimethylimidazole-4-sulfonyl chloride (DMISC), using multi-stage mass spectrometry [31]. In our previous study, a different pre-glucuronidation derivatization method using 4-methoxypropranolol (4-MeOP) as a substrate was employed to investigate the regioselectivity, and the results suggested that the aliphatic hydroxy group was glucuronidated when the aromatic hydroxy group was blocked [28].

In this study, we conducted a reaction phenotyping of 5-OHP and 7-OHP with 19 human UGTs, following a previously established protocol [28]. The presence of hydroxypropranolol glucuronides was confirmed in both in vitro and in vivo experiments, by comparing the results with human liver microsomes (HLMs) incubations and urine samples [22]. Moreover, the regioselectivity of 5- and 7-OHP glucuronidation was investigated using both pre-glucuronidation derivatization (using methoxypropranolols as substrates) and post-glucuronidation derivatization (using DMISC as derivatization reagent) methods.

## 2. Results

### 2.1. Identification of 4, 5- and 7-OHPGs

In this study, 4-, 5- and 7-OHP were incubated with HLMs. The HLM samples mentioned in this section were incubated with substrates for 4 h, and they should be distinguished from the HLM samples that were used for derivatization in Section 2.3.2 (details in Section 4.4). These in vitro samples and urine samples were analyzed by LC-MS/MS and compared with each other to determine which of these hydroxypropranolols are glucuronidated in the human body. A urine sample that was collected 17.6 h after oral propranolol administration was used for comparison as the amounts of hydroxypropranolol glucuronides in this test sample were found to be relatively higher than those of the other urine samples (details in Section 4.5). Furthermore, HLMs were incubated with (*R*)-propranolol in the presence of both NADPH and UGPGA as cofactors. This experiment allowed for the generation of the (*R*)-forms of 4-, 5-, and 7-OHP, which were subsequently glucuronidated to generate the corresponding (*R*)-OHPG metabolites. This sample was used for assigning the individual (*R*)-OHPG isomers in urine samples. By identifying the (*R*)-diastereomer, the remaining diastereomers were determined to be the (S)-forms.

**Figure 1:**
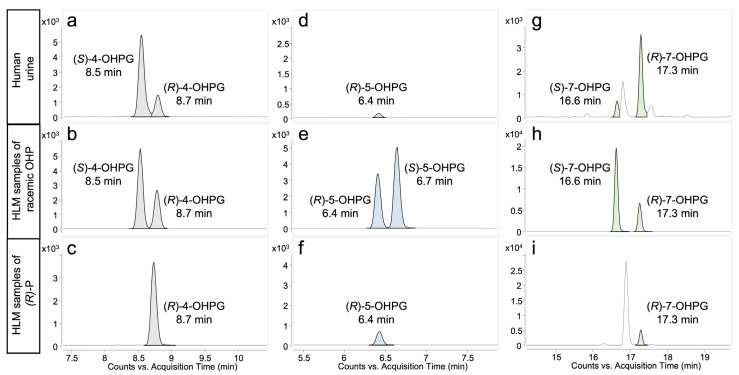
Chromatograms (LC-QQQ-MS/MS) of 4-, 5- and 7-hydroxypropranolol diastereomeric glucuronides in urine sample and 4-h HLM incubations. The displayed ion transitions are *m/z* 452→116 (4-OHPG), 452→98 (5-OHPG), and 452→276 (7-OHPG); (*R*)-P, (*R*)-propranolol; OHP, hydroxypropranolol; OHPG, hydroxypropranolol glucuronide. (**a**,**d**,**g**) refer to the analysis of urine sample (collected 17.6 h post administration); (**b**,**e**,**h**) are from incubation of HLM with (±)-4-hydroxypropranolol, (±)-5-hydroxypropranolol and (±)-7-hydroxypropranolol, respectively; (**c**,**f**,**i**) were from HLM incubated with (*R*)-propranolol, UDPGA and NADPH.

**Figure 2:**
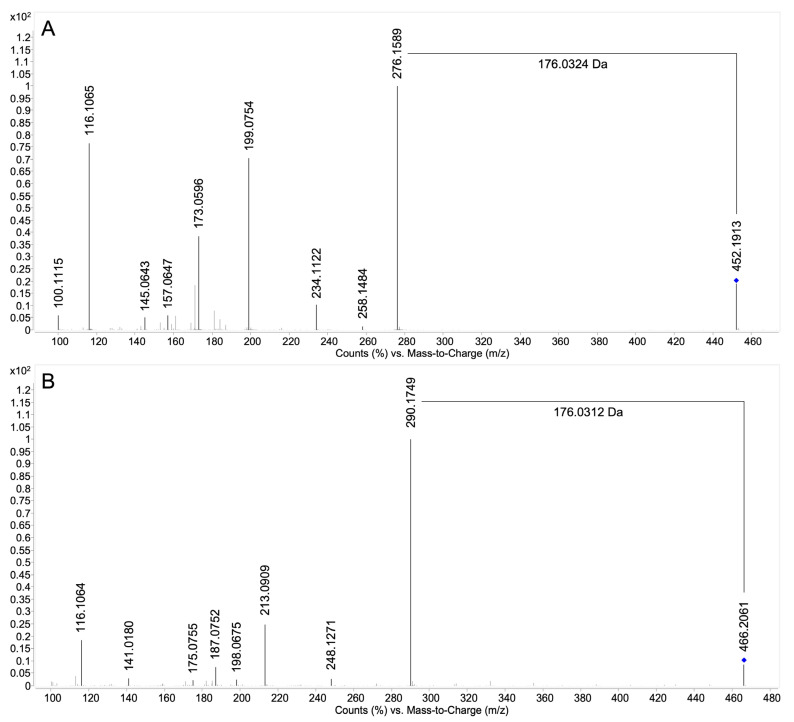
Product ion spectra (LC-QTOF-MS/MS) of (*R*)-5-hydroxypropranolol glucuronide and 5-methoxypropranolol glucuronide obtained from HLM incubations. (**A**) 5-Hydroxypropranolol glucuronide, C_22_H_29_NO_9_, [M+H]^+^ theor. = 452.1915, [M+H]^+^ exp. = 452.1913, ∆*m/z* = −0.44 ppm, collision energy 20 eV; (**B**) 5-methoxypropranolol glucuronide, C_23_H_31_NO_9_, [M+H]^+^ theor. = 466.2072, [M+H]^+^ exp. = 466.2061, ∆*m/z* = −2.36 ppm, collision energy 20 eV. The blue diamond indicates the precursor ion.

**Figure 3:**
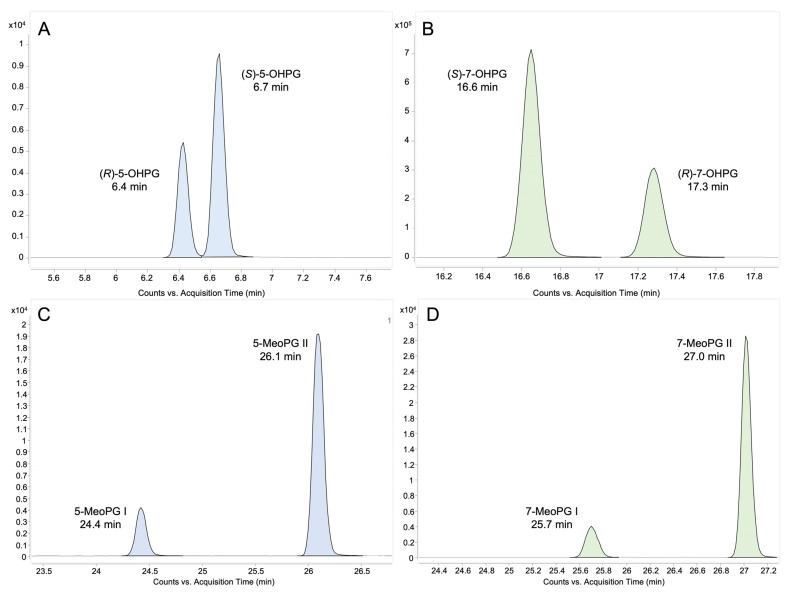
Chromatograms (LC-QQQ-MS/MS) of 5-/7-hydroxypropranolol diastereomeric glucuronides (**A**,**B**) and 5-/7-methoxypropranolol diastereomeric glucuronides (**C**,**D**) obtained from UGT1A9 enzyme bags samples. The displayed ion transitions are *m/z* 452→98 (5-OHPG), *m/z* 452→276 (7-OHPG) and *m/z* 466→290 (5- and 7-MeOPG). 5-/7-OHPG, 5-/7-hydroxypropranolol glucuronide; 5-/7-MeOPG, 5-/7-methoxypropranolol glucuronide.

**Figure 4:**
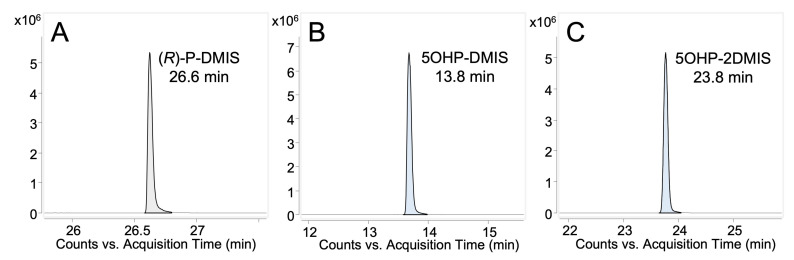
Extracted ion chromatograms obtained from MS full-scan analysis (LC-QTOF-MS) of DMIS derivatives. (**A**) (*R*)-P-DMIS, [M+H]^+^ *m/z* 418, mono-DMIS derivative of (*R*)-propranolol; (**B**) 5OHP-DMIS, [M+H]^+^ *m/z* 434, mono-DMIS derivative of 5-hydroxypropranolol; (**C**) 5OHP-2DMIS, [M+H]^+^ *m/z* 592, bis-DMIS derivative of 5-hydroxypropranolol.

Figure 1 illustrates the presence of two diastereomeric glucuronides in both urine and HLM samples for the testing of 4-OHP and 7-OHP. By comparison with (*R*)-propranolol incubation (Figure 1c) (*R*)-4-OHPG was assigned to the peak eluting at 8.7 min, thus (*S*)-4-OHPG was eluted at 8.5 min (Figure 1a,b). Similarly, (*S*)-7-OHPG and (*R*)-7-OHPG were assigned to the peaks eluting at 16.6 min and 17.3 min, respectively (Figure 1g–i). For 5-OHP, HLMs incubation resulted in two glucuronides but only the (*R*)-diastereomer was found in the urine sample (Figure 1d–f). (*S*)-5-OHPG and (*R*)-5-OHPG were eluted at 6.4 min and 6.6 min, respectively.

Targeted MS/MS analysis was performed on each diastereomer for the additional characterization by high-resolution accurate mass spectrometry on a quadrupole time of flight (QTOF) instrument. Figure 2A shows the product ion spectrum of (*R*)-5-OHPG. The loss of 176.0324 Da indicated the loss of the glucuronic acid moiety. The product ion spectra of all diastereomers of 4-, 5- and 7-OHPG were highly similar. Therefore only (*R*)-5-OHPG is shown here, while the product ion spectra of 4- and 7-OHPG are shown in Supplementary Figure S1.

### 2.2. Glucuronidation of 5- and 7-OHP by 19 UGT Enzyme Bags

In our previous study, activity assays of propranolol and 4-OHP with 19 UGTs were performed and five active UGTs (UGT1A7, UGT1A9, UGT1A8, UGT1A10, and UGT2A1) were identified [28]. In this study, a similar experiment was conducted on 5-OHP and 7-OHP to determine the human UGTs responsible for their metabolism, as their glucuronides were observed in both human urine samples and HLM incubations. Enzyme bags for the 19 human UGTs were prepared as described in the previous study [28] and incubated with 5-OHP and 7-OHP, respectively.

Glucuronidation of 5-OHP was observed for eight UGTs, namely UGT1A1, 1A3, 1A7, 1A8, 1A9, 1A10, 2A1, and 2A2, with varying degrees of activity. Additionally, these eight UGTs, along with UGT1A6, were also involved in the glucuronidation of 7-OHP. By comparison of the retention times, the configurations of 5- and 7-OHP diastereomeric glucuronides were assigned based on the results from Figure 1f,i, as shown in Figure 3A,B. A summary of the activity of 19 UGTs towards racemic 5-OHP and 7-OHP is presented in Table 1, with activity determined by the chromatographical peak areas of 5- and 7-OHPGs, respectively.

### 2.3. Determination of the Regioselectivity of 5- and 7-OHP Glucuronidation

As stated in the introduction, this study utilized two separate methods to determine the position of the glucuronic acid moiety in hydroxypropranolol glucuronides based on previous studies [28,31].

#### 2.3.1. Pre-Glucuronidation Derivatization

The purpose of this experiment was to investigate whether the aliphatic hydroxy group undergoes glucuronidation when the aromatic hydroxy group is methylated. In accordance with our previous study [28], the glucuronidation of 5- and 7-methoxypropranolol (5- and 7-MeOP) was carried out using the nine UGTs that were active in the glucuronidation of 5- and 7-OHP, applying the enzyme bags method. Due to the absence of enantiopure 5- and 7-MeOP, the configuration of the methoxypropranolol glucuronide diastereomers could not be assigned. They are therefore labeled as glucuronide I and II. As illustrated in the Figure 3C,D, 5-methoxypropranolol glucuronide I (5-MeOPG I) and II (5-MeOPG II) were eluted at 24.4 min and 26.1 min, while 7-methoxypropranolol glucuronide I (7-MeOPG I) and II (7-MeOPG II) were eluted at 25.7 min and 27.0 min, respectively. Table 2 provided a summary of the activity of the UGTs that were active in glucuronidation towards racemic 5- and 7-MeOP, as indicated by the chromatographical peak areas of the methoxypropranolol glucuronides.

Additional MS/MS analysis was performed for the characterization of 5- and 7-MeOPGs with high-resolution accurate mass spectrometry. The product ion spectra of 5- and 7-MeOPGs were highly similar, therefore only 5-MeOPG is shown in Figure 2B. The loss of 176.0312 Da indicated the loss of the glucuronic acid moiety. The product ion spectra of 4- and 7-MeOPG are shown in Supplementary Figure S2.

#### 2.3.2. Post-Glucuronidation Derivatization

In a previous study, Salomonsson et al. reported that both aromatic-linked and aliphatic-linked 4-OHPGs were formed under in vitro conditions, using multi-stage mass spectrometry after derivatization with DMISC [31]. Thus, DMISC was also employed to differentiate 5- and 7-OHPG regio-isomers in this study.

Following incubation of 5- and 7-OHP with HLMs and UDPGA for 24 h, the solutions were subjected to derivatization using DMISC. For comparison, propranolol and 5-/7-OHP were also incubated with DMISC, and all derivatized samples were analyzed by full-scan MS and targeted MS/MS with LC-QTOF-MS. Additionally, 4-OHP was incubated with HLMs, and the generated 4-OHPG was derivatized with DMISC as well. The 1,2-dimethylimidazole-4-sulfonyl (DMIS) derivatives of 4-OHP and 4-OHPG were also analyzed for additional confirmation of this protocol developed by Salomonsson et al. [31].

##### DMIS Derivatives of Propranolol and Hydroxypropranolols

**Figure 5:**
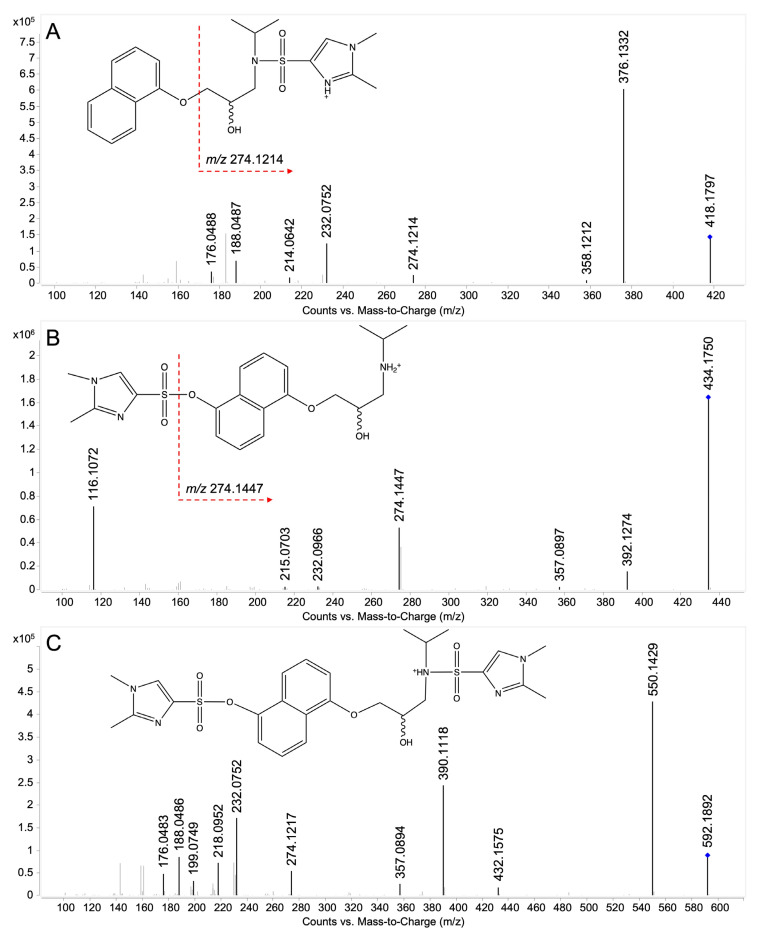
Product ion spectra of (*R*)-propranolol and 5-hydroxypropranolol DMIS derivatives. (**A**) (*R*)-propranolol-DMIS, C_21_H_27_N_3_O_4_S, corresponding to the peak in Figure 4A, collision energy 20 eV; (**B**) 5-hydroxypropranolol-DMIS, C_21_H_27_N_3_O_5_S, corresponding to the peak in Figure 4B, collision energy 20 eV; (**C**) 5-hydroxypropranolol-2DMIS, C_26_H_33_N_5_O_7_S_2_, corresponding to the peak in Figure 4C, collision energy 30 eV. The dashed red arrows indicated the postulated position of cleavage. The blue diamond indicates the precursor ion.

The full-scan analysis showed the molecular ion [M+H]^+^ of the DMIS derivatives of propranolol and 5-OHP, as illustrated in Figure 4. Previous studies indicated that DMISC is phenolic selective but that it can also react with secondary amine groups [31,32,33]. Similar to the findings of Salomonsson et al. [31], propranolol yielded a DMIS derivative. Due to a lack of a phenolic hydroxy group, this was assigned to the *N*-derivatized propranolol. In the case of 5-OHP, two derivatives were detected, i.e., *O*-DMIS (5OHP-DMIS, Figure 4B) and *N,O*-bis-DMIS (5OHP-2DMIS, Figure 4C). Targeted MS/MS analysis was performed on the precursor corresponding to protonated propranolol-DMIS (P-DMIS, *m/z* 418) or 5-hydroxypropranolol-DMIS (5OHP-DMIS, *m/z* 434), as shown in Figure 5A,B. All the fragments of (*R*)-propranolol-DMIS and 5OHP-DMIS are summarized in Table 3 and Table 4, respectively.

In P-DMIS (Table 3), ions *m/z* 376.1332 and *m/z* 358.1212 are generated from the precursor ion *m/z* 418.1797 by a loss of the isopropyl group (-C_3_H_6_, 42 Da) and water (-H_2_O, 18 Da). The ion *m/z* 274.1214 may be explained by the loss of the hydroxy naphthyl ring (-C_10_H_8_O, 144 Da) from the precursor ion *m/z* 418.1797. The additional loss of the isopropyl group and water results in *m/z* 232.0752 and *m/z* 214.0642. In the propranolol-DMIS derivative, the basic dimethylimidazole ring may also get protonated as reported in the previous study [31], generating charged fragments *m/z* 188.0487 and *m/z* 176.0488 that still contain the DMIS moiety.

In contrast, the fragmentation pattern of 5OHP-DMIS is distinctly different (Figure 5B). As shown in Table 4, the ion *m/z* 392.1274 derived from the protonated quasi-molecular ion [M+H]^+^ (*m/z* 434.1750) by losing the isopropyl group. Fragment *m/z* 357.0897 indicates a loss of 77 Da from [M+H]^+^, which is suggested to result from the combined elimination of water (-H_2_O, 18 Da) and isopropylamine (-C_3_H_9_N, 59 Da). As reported in the study about DMIS derivatives of 4-OHP by Salomonsson et al. [31], the DMIS moiety is suggested to be lost as a neutral sulfone, resulting in the fragment *m/z* 274.1447 with the charge on the naphthyl ring (Figure 5B). The subsequent loss of the isopropyl group (-C_3_H_6_, 42 Da) or isopropylamine generates *m/z* 232.0966 or *m/z* 215.0703, respectively. Fragment *m/z* 116.1072 indicates the intact aminoalcoholic chain without DMIS attached. Notably, in the high-resolution mass spectrum, the ions *m/z* 274.1447 and *m/z* 232.0966 have different digits after the decimal point, compared with the ions *m/z* 274.1214 and *m/z* 232.0752 obtained from propranolol-DMIS (Figure 5A) indicating that their structures were different and therefore can be used for identification given a mass resolving power over 10,000.

The MS full-scan analysis of the DMIS derivatives of 5-OHP revealed that DMIS may attach to both the phenolic and the secondary amine group when a derivatization reagent is added in excess (Figure 4C). To further investigate this, a targeted MS/MS analysis of the corresponding 2-fold derivatized 5-OHP (precursor *m/z* 592) was performed. The resulting spectra (Figure 5C) show combined fragmentation pathways from propranolol-DMIS and 5OHP-DMIS. Table 5 shows the fragments of the bis-DMIS derivative of 5-OHP. Two DMIS moieties were inferred to be attached based on the detection of ion *m/z* 550.1429, which was generated by the loss of the isopropyl group (-C_3_H_6_, 42 Da) from the precursor ion *m/z* 592.1892. The loss of aromatic-linked DMIS moiety from the precursor ion results in the ion *m/z* 432.1575. The loss of aromatic-linked DMIS moiety from ion *m/z* 550.1429 results in the ion *m/z* 390.1118. Comparison with Figure 5A indicates that the ions *m/z* 218.0952, 188.0486, and 176.0483 are fragments where DMIS was attached to the amine group. Similarly, the ion *m/z* 357.0894 is a fragment where DMIS is attached to the phenolic group. The ion *m/z* 199.0749 is considered as protonated precursor ion *m/z* 592.1892 losing both DMIS moieties, isopropylamine (-C_3_H_9_N, 59 Da) and water (-H_2_O, 18 Da). The ions *m/z* 274.1217 and *m/z* 232.0752 are supposedly fragments where DMIS was amine-linked because the digits after the decimal point were closer to the fragment ions produced by protonated propranolol-DMIS (Figure 5A).

Full-scan and MS/MS analyses were performed on the DMIS derivatives of 4-OHP (mono and bis-DMIS derivatives, Supplementary Figures S3 and S4) and 7-OHP (Supplementary Figures S5 and S6). The resulting fragmentation patterns were highly similar to those of the 5-OHP DMIS derivatives. Furthermore, additional full-scan MS was conducted in search of aliphatic-linked DMIS derivatives of propranolol and hydroxypropranolols, but no such derivatives were detected. These results are in line with earlier reports that derivatization with DMISC does not occur in the aliphatic hydroxy groups [31,32,33].

##### DMIS Derivatives of Hydroxypropranolol Glucuronides

**Figure 6:**
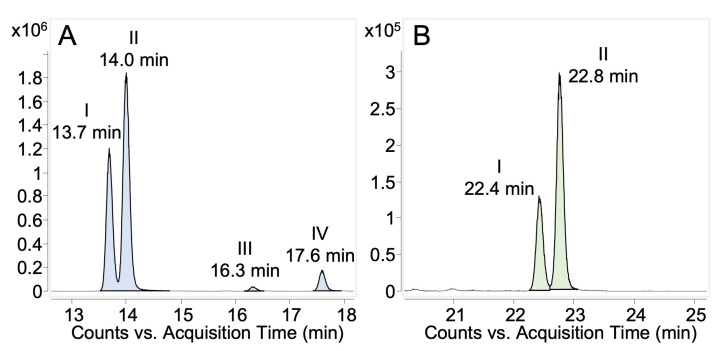
Extracted ion chromatograms from MS full-scan (LC-QTOF-MS) of 5- and 7-hydroxypropranolol glucuronides DMIS derivatives obtained from 24-h HLM incubations. (**A**) 5-OHPG-DMIS I, II, III, and IV, [M+H]^+^ *m/z* 610, 5-hydroxypropranolol glucuronide DMIS derivatives; (**B**) 7-OHPG-DMIS I and II, [M+H]^+^ *m/z* 610, 7-hydroxypropranolol glucuronide DMIS derivatives.

The full-scan LC-MS analysis of the 24-h HLM incubations of 5-OHP or 7-OHP shows chromatographic peaks at *m/z* 610 for 5- and 7-OHPG DMIS derivatives (Figure 6). Four DMIS derivatives of 5-OHPG were observed and indicated as 5-OHPG-DMIS I, II, III, and IV with retention times of 13.7 min, 14.0 min, 16.3 min, and 17.6 min (Figure 6A). In Figure 6B, two DMIS derivatives of 7-OHPG were found and indicated as 7-OHPG-DMIS I and II with retention times of 22.4 min and 22.8 min.

**Figure 7:**
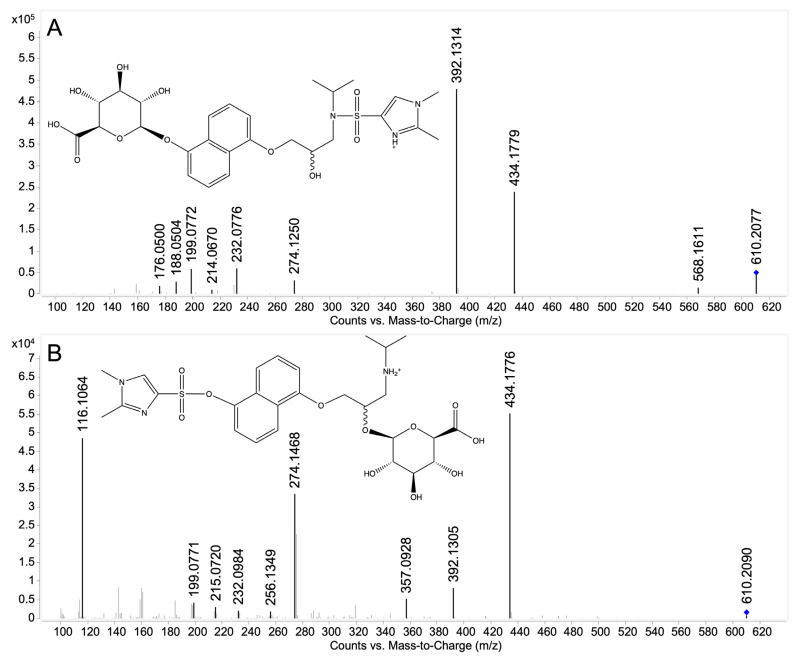
Product ion spectra of 5-hydroxypropranolol glucuronides DMIS derivatives obtained from HLM incubations. (**A**) 5-hydroxypropranolol glucuronide-DMIS I, C_27_H_35_N_3_O_11_S, [M+H]^+^ theor. = 610.2065, [M+H]^+^ exp. = 610.2077, Δ*m/z* = 1.97 ppm, collision energy 30 eV; (**B**) 5-hydroxypropranolol glucuronide-DMIS III, C_27_H_35_N_3_O_11_S, [M+H]^+^ theor. = 610.2065, [M+H]^+^ exp. = 610.2090, Δ*m/z* = 4.10 ppm, collision energy 30 eV. The blue diamond indicates the precursor ion. Postulated structures of the obtained fragments were demonstrated in supplementary Figures S7 and S8.

Targeted MS/MS analysis was conducted on 5-OHPG-DMIS I, II, III, and IV. Two pairs of similar product ion spectra of 5-OHPG-DMIS (I and II as well as III and IV) were obtained. Therefore, only the spectra of 5-OHPG-DMIS I and III are displayed in Figure 7. In Figure 7A, the ion *m/z* 568.1611 indicated the loss of the isopropyl moiety from the protonated precursor *m/z* 610.2077. The loss of the sugar moiety from *m/z* 610.2077 resulted in *m/z* 434.1779. The *ion* m/z 392.1314 is derived from the additional loss of an isopropyl group compared to fragment ion *m/z* 434.1779. The ions *m/z* 214.0670, 188.0504, and 176.0500 are produced from the fragments where the DMIS moiety is attached to the amine group. Additionally, the ions *m/z* 274.1250 and 232.0776 are considered fragments where DMIS was amine-linked because their accurate mass is closer to the fragment ions produced by propranolol-DMIS (*m/z* 418, Figure 5A). Therefore, it was concluded that 5OHPG-DMIS I and II are phenolic-linked glucuronide derivatives since DMIS was attached to the amine group.

Conversely, as shown in Figure 7B, the ions *m/z* 357.0928 and 215.0720 are derived from the fragments with DMIS attached to the phenolic group. Again, the ion *m/z* 392.1305 also results from the additional loss of the isopropyl group from *m/z* 434.1779. Besides, the ions *m/z* 274.1468 and 232.0984 are identified as fragments where DMIS is phenolic-linked, considering that their accurate mass is closer to that of the fragment ions generated from protonated 5OHP-DMIS (Figure 5B). Therefore, 5-OHPG-DMIS III and IV were considered aliphatic-linked glucuronides since the DMIS was attached to the phenolic group.

Product ion spectra of 7-OHPG-DMIS I and II (Supplementary Figure S9) showed a similar fragmentation pattern as 5OHPG-DMIS I (Figure 7A). Suggested structures of fragments derived from 7-OHPG-DMIS I and II are highly similar to those derived from 5-OHPG-DMIS I. Further details are shown in Supplementary Figure S7. Therefore, the two derivatives of 7-OHPG were assigned as amine-linked DMIS derivatives. The diastereomeric glucuronides of 7-OHP were considered to be aromatic-linked glucuronides since the glucuronic acid occupied the phenolic hydroxy group which therefore has not been amenable for derivatization.

As a positive control and additional confirmation of the feasibility of the post-glucuronidation derivatization method adapted from the previous study [31], the same derivatization reaction was conducted on (*S*)-4-OHPG (derived from HLM samples) and the samples were analyzed by full-scan MS and targeted MS/MS. The chromatograms and product ion spectra of (*S*)-4-OHPG-DMIS I and II are shown in Supplementary Figure S10 and S11. The product ion spectra indicated that (*S*)-4-OHPG-DMIS I and II are aromatic-linked glucuronide and aliphatic-linked glucuronide after comparing their fragmentation pattern with Figure 7. Suggested structures of fragments derived from (*S*)-4-OHPG-DMIS I and II are highly similar to those derived from 5-OHPG-DMIS I and III. Further details are shown in Supplementary Figures S7 and S8. Those results are in line with the previous study [31] that both aromatic and aliphatic-linked glucuronides of 4-OHP are able to be generated under in vitro conditions.

The full-scan and MS/MS analysis were also conducted on the DMIS derivatives of propranolol glucuronides. Two diastereomeric propranolol glucuronide-DMIS were observed, eluting at 20.5 min and 21.1 min (Supplementary Figure S12). The MS/MS analysis of the protonated propranolol glucuronide-DMIS (*m/z* 594) revealed a prominent ion at *m/z* 418, indicating the loss of 176 Da, representing the glucuronide moiety, from the protonated precursor ion. The subsequent fragmentation pattern closely matched the MS/MS results obtained for propranolol-DMIS (as depicted in Figure 5A), and these findings are also displayed in Supplementary Figure S13. The postulated structures of the obtained fragments were demonstrated in Supplementary Figure S14. Collectively, these results suggest that the DMIS moiety is attached to the same secondary amine group in both propranolol and propranolol glucuronide molecules.

## 3. Discussion

Since the 1980s, mono-hydroxylated metabolites of propranolol, specifically 4-OHP, 5-OHP, and 7-OHP, have been detected in both human and rat urine [21,34]. Among these, 4-OHP has long been recognized as the primary phase I metabolite of propranolol in humans and laboratory animals [34,35,36]. Together with sulfation, glucuronidation is an important elimination pathway of 4-OHP [37]. However, investigations into the phase II metabolism of the other two monohydroxylated metabolites, 5-OHP and 7-OHP, are still missing In this study, we conducted an investigation into the glucuronidation of 5-OHP and 7-OHP and compared the results with the glucuronidation of 4-OHP [28].

The glucuronidation of all three monohydroxylated phase I metabolites of propranolol was found to be carried out in the human body, as evidenced by the combined analysis of hydroxypropranolol glucuronidation in both in vivo (human urine) and in vitro (HLMs) samples (Figure 1). The results of reaction phenotyping of 5- and 7-OHP by 19 UGTs (Table 1) indicated the involvement of nine UGTs in the glucuronidation of hydroxypropranolols. Importantly, the most active isoforms for the glucuronidation of 5- and 7-OHP were found to be the same as those identified for 4-OHP in a previous study: UGT1A7, UGT1A8, UGT1A9, and UGT2A1 [28]. Furthermore, four additional UGTs, namely UGT1A1, UGT1A3, UGT1A10, and UGT2A2, were found active in the glucuronidation of 5-OHP. Moreover, UGT1A6 also exhibited certain glucuronidation activity towards 7-OHP, in addition to the aforementioned eight UGTs for 5-OHP. The remaining UGTs exhibited no detectable glucuronidation activity towards 5- or 7-OHP. This lack of activity may be attributed to the selectivity of different UGT enzymes for the same substrate. Such selectivity might arise from the fact that these inactive UGTs have different binding conformations to 5- or 7-OHP due to the difference in protein sequences compared with the active UGTs. In a previous study [28], we discovered that the four UGTs metabolizing 4-OHP (UGT1A7, UGT1A8, UGT1A9, and UGT2A1) share a loop with a highly similar sequence at the substrate binding site. This loop is believed to play a crucial role in the binding conformation of 4-OHP to the four UGTs, thereby facilitating the glucuronidation reaction. Further in silico studies on the binding of 5- or 7-OHP to UGTs could be carried out for a more comprehensive understanding of the structural and functional aspects influencing glucuronidation activity. It is worth noting that in both urine and HLMs samples (Figure 1a,b), (*S*)-4-OHPG is observed as the most abundant isomer. However, the results for 5- and 7-OHPG present a contradictory picture. Specifically, in the case of 5-OHPG, Figure 1d illustrates that only (*R*)-5-OHPG is detected in the urine sample, with no observation of (*S*)-5-OHPG, or the produced quantity being below the limit of detection. In contrast, in the HLMs sample, (*S*)-5-OHPG is the dominant metabolite compared to (*R*)-5-OHPG. A similar contradictory outcome was also observed for 7-OHPG, with (*R*)-7-OHPG being the primary metabolite in the urine sample (Figure 1g), while the reverse is true in the HLMs sample (Figure 1h).

Previous research has consistently indicated that (*R*)-propranolol is the preferred substrate for hydroxylation at the 5- and 7-positions [21,22,36]. Given these findings, it is reasonable to infer that the observed preference for (*R*)-5-OHP in urine (Figure 1d) and (*R*)-7-OHP (Figure 1g) may be a consequence of the stereoselective 5- and 7-hydroxylation of (*R*)-propranolol, rather than the stereoselective glucuronidation of (*R*)-5- and (*R*)-7-OHP. Furthermore, it is important to note that 4-hydroxylation of propranolol has been found to exhibit either minimal or no stereoselectivity in both urine samples and CYP2D6 incubations [21,22]. Therefore, the stereoselectivity observed in the glucuronidation of 4-OHP remains consistent in both urine and HLMs samples (Figure 1a,b). These results emphasize the importance of phase I metabolism in explaining the variations between in vitro and in vivo data when studying the phase II metabolism of chiral drugs. Recognizing this factor is crucial when establishing correlations between in vitro and in vivo data for drug metabolism and pharmacokinetics studies.

Interestingly, an additional peak eluting at approximately 16.9 min was observed in both urine and HLMs incubation with (*R*)-propranolol (Figure 1g,i). Tandem MS analysis (Supplementary Figure S15) revealed that the spectra exhibited the same fragmentation pattern as shown in 5-OHP (Figure 2A). This suggested that an additional glucuronide metabolite of hydroxypropranolol was generated. One possible explanation is that an extra glucuronide may have been formed from 4-, 5-, or 7-OHP in addition to the glucuronides depicted in Figure 1. However, no peaks corresponding to glucuronides were detected at the same retention time in either HLM incubation or enzyme bags with 4-, 5-, or 7-OHP as substrates (Figure 1 and Figure 3). Hence, it is unlikely that the peak at 16.9 min is related to the glucuronidation of 4-, 5-, or 7-OHP. The other possibility is the potential formation of another hydroxypropranolol during the hydroxylation of propranolol, apart from 4-, 5-, and 7-OHP. Previous studies have detected 2-OHP in both rat and human samples, albeit in minor quantities [21]. Further investigations will be needed to determine whether the additional peak corresponds to 2-OHP glucuronide or not.

Identifying the precise site of conjugation in the glucuronidation of hydroxypropranolols poses a significant challenge, primarily due to the presence of two active hydroxy groups. A prior study has demonstrated that the glucuronidation site of 4-OHP is the aromatic hydroxy group [30]. However, it has been confirmed that the aliphatic hydroxy group on the side chain is also active in propranolol glucuronidation [28]. Consequently, it appears improbable that the glucuronidation of hydroxypropranolols is limited to the aromatic hydroxy group. Tandem mass spectrometry analysis serves as a practical method for confirming the structure because it imposes fewer constraints regarding sample purity and concentration compared to NMR. However, the direct fragmentation of glucuronides results in the loss of the glucuronic acid moiety, making it challenging to pinpoint the exact glucuronidation site. To address this issue, two methods were employed in the current study.

In this study, pre-glucuronidation derivatization was initially performed to investigate the glucuronidation position. 5- and 7-MeOP were incubated with the UGTs that were previously identified to be active in the glucuronidation of 5- and 7-OHP. The rationale behind this method is that the phenolic group is occupied by a methyl group, so if the glucuronides were aromatic-linked metabolites, no glucuronides would be produced. However, UGT1A1, 1A3, 1A7, 1A9, and 2A1 still exhibited varying abilities to glucuronidate the side-chain hydroxy group, as listed in Table 2. This result aligned with the metabolism of 4-methoxypropranolol by UGT1A9 and UGT2A1 in our previous study [28]. Those results indicated that glucuronidation can occur at the aliphatic hydroxy group under certain circumstances when the aromatic hydroxy group is blocked.

Thus, a direct method for characterization of the hydroxypropranolol glucuronides is required. As reported by Thompson et al. [30], a post-glucuronidation derivatization was developed. GC-MS/MS analysis of diazomethane derivatives indicated that the glucuronides of 4-OHP were aromatic-linked in human and dog urine. However, it is important to note that diazomethane is a highly reactive and potentially hazardous compound, requiring proper precautions and protective equipment during handling [38]. As an alternative, DMISC has been developed as a new derivatization reagent for phenolic compounds and has been successfully employed in the derivatization of various compounds, including propofol, hydroxypyrene, naphthol, estrone, and others [33,39]. Furthermore, DMISC has been effectively utilized to confirm the glucuronidation position of 4-hydroxypropranolol glucuronides [31]. Thus, DMISC was employed to differentiate the regio-isomers of 5- and 7-OHP glucuronides in this study. DMISC was found to selectively react with phenolic hydroxy groups and amine groups but not aliphatic hydroxy groups [31,33]. This observation was further supported by the MS full scan analysis, which showed the absence of bis-DMIS derivatives of propranolol or tris-DMIS derivatives of hydroxypropranolol.

Four DMIS derivatives of 5-OHPG were detected in the MS full-scan analysis (5OHPG-DMIS I, II, III, and IV, respectively, Figure 6A). Based on the fragmentation results of propranolol-DMIS and hydroxypropranolol-DMIS derivatives, 5OHPG-DMIS I and II were found to be derived from aromatic-linked glucuronides, as evidenced by the presence of ions at *m/z* 374, *m/z* 214, *m/z* 188, *m/z* 274.1250 and *m/z* 232.0774 in the product ion spectra (Figure 7A), indicating the presence of amine-linked DMIS derivatives. On the other hand, 5OHPG-DMIS III and IV were identified as derivatives generated from aliphatic-linked glucuronides, as indicated by the presence of ions at *m/z* 357, *m/z* 319, *m/z* 215, *m/z* 274.1468 and *m/z* 232.0984 which are indicative of aromatic-linked DMIS derivatives (Figure 7B).

Four DMIS derivatives of 5-OHPG were observed in the 24-h incubated HLM sample after the derivatization. However, in the urine sample and 4-h incubated HLMs sample (Figure 1d,e), at most two 5-OHPGs were observed, which raises an intriguing question. To gain further insights, additional LC-QQQ-MS/MS analysis was conducted on the 24 h incubated HLMs sample prior to derivatization with DMISC. In this analysis, the two 5-OHPGs that were previously identified were intentionally excluded by cutting off before the MS analysis based on their retention time (go to the waste), in order to eliminate the influence of their high signals. Interestingly, this led to the observation of two additional glucuronides, eluted at 15.0 min and 16.4 min (Supplementary Figures S16 and S17). However, it is worth noting that these glucuronides exhibited much lower abundance than the two 5-OHPGs previously identified. This finding suggests that these newly identified glucuronides may be minor metabolites.

Therefore, it is hypothesized that the two less abundant 5OHPG-DMIS derivatives, 5OHPG-DMIS III and IV (Figure 6A), could have originated from these two additional glucuronides, as they were similarly less abundant. Consequently, the two additional 5-OHPGs observed in HLM 24-h incubations are believed to be the aliphatic-linked hydroxypropranolol glucuronides, making the previously identified 5-OHPGs in urine, 4-h incubated HLM sample, and enzyme bags samples aromatic-linked hydroxypropranolol glucuronides.

The same LC-QQQ-MS/MS analysis was conducted on urine samples, 4-h incubated HLMs samples, and enzyme bag samples. Such extra glucuronides, eluted at 15.0 min and 16.4 min, were detected in enzyme bag samples, but not in the urine samples and 4-h incubated HLMs samples. The possible explanation is that the HLMs samples used for derivatization were incubated with a higher concentration of 5-OHP and a prolonged incubation time of 24 h. Similarly, the enzyme bag samples were incubated for a long period of 15 h. Under such reaction conditions, it is possible that more glucuronides may be generated. However, in vivo, metabolic studies have suggested that 5-OHP accounts for only 7% to 11% of the total monohydroxylated propranolol [21,34], which is a relatively low amount to produce additional aliphatic-linked glucuronides. Therefore, no extra 5-OHPG was found in urine. It is hypothesized that the 5-OHPG observed in urine (Figure 1d) is related to the amine-linked DMIS derivative (Figure 6A) and is considered to be aromatic-linked hydroxypropranolol glucuronide. Due to the complexity of urine samples, derivatization with DMISC gave more complicated results to interpret. Further derivatization experiments with isolated 5-OHPGs from urine samples will be needed for the confirmation of the inference above.

Results from full-scan MS and MS/MS analysis on 7-OHPG-DMIS (Figure 6B and Supplementary Figure S9) indicated that the two DMIS derivatives of 7-OHPG were amine-linked. The diastereomeric glucuronides of 7-OHP were considered to be aromatic-linked glucuronides since the aromatic hydroxy group is occupied by glucuronic acid and the DMIS moiety is thereby attached to the secondary amine group. Moreover, full-scan MS and MS/MS analysis on 4OHPG-DMIS (Supplementary Figures S10 and S11) showed that both aromatic and aliphatic-linked glucuronides were produced in HLM samples, which is in line with the previous study [31].

In summary, the findings from both pre-and post-derivatization studies indicate that the aliphatic hydroxy group of hydroxypropranolol can undergo glucuronidation in principle. However, under the in vivo environment after the administration of propranolol, it appears to be less favorable compared to hydroxynaphthyl glucuronidation. On the other hand, in vitro studies with high concentrations of hydroxypropranolols and extended incubation time demonstrated the possibility of aliphatic-linked glucuronidation. Overall, it can be concluded that under physiological conditions, hydroxynaphthyl glucuronidation is the preferred glucuronic pathway for the elimination of hydroxypropranolols.

## 4. Materials and Methods

### 4.1. Chemicals and Reagents

Tris, potassium chloride, glycerol, and Triton X-100 were from Carl Roth GmbH + Co. KG (Karlsruhe, Germany); uridine diphosphate glucuronic acid (UDPGA), (±)-propranolol, (*R*)-propranolol and (±)-4-hydroxypropranolol hydrochloride were from Sigma-Aldrich (Taufkirchen, Germany); (±)-5-hydroxypropranolol was from Cayman Chemical Company (Ann Arbor, MI, USA); (*S*)-4-hydroxypropranolol was from Santa Cruz Biotechnology Inc. (Dallas, TX, USA); pooled human liver microsomes were from Corning Inc. (Somerville, MA, USA); the NADPH regeneration system was from Promega Corporation (Madison, WI, USA); (±)-5-methoxypropranolol, (±)-7-methoxypropranolol and (±)-7-hydroxypropranolol were synthesized in-house as described previously [28,40]. Ammonium hydrogen carbonate (NH_4_HCO_3_) was from VWR International GmbH (Darmstadt, Germany); 1× PBS was from VWR Chemicals. LLC (Solon, OH, USA); 1,2-dimethylimidazole-4-sulfonyl chloride (DMISC) was from Sigma-Aldrich (St. Louis, MO, USA); acetone (GC-MS grade) was from Merck KGaA (Darmstadt, Germany); sodium carbonate anhydrous was from Ferak Berlin GmbH (Berlin, Germany).

### 4.2. Preparation of Compound Solutions

All substrates, i.e., (±)-propranolol, (*R*)-propranolol, (±)-4-hydroxypropranolol, (*S*)-4-hydroxypropranolol (±)-5-hydroxypropranolol, (±)-7-hydroxypropranolol, (±)-4-methoxypropranolol, (±)-5-methoxypropranolol, and (±)-7-methoxypropranolol, were prepared as 10 mM stock solutions with methanol of LC-MS grade. These stock solutions were diluted to a final concentration of 1 mM in the metabolic reaction system containing either HLMs or enzyme bags. For DMISC derivatization, all substrate stock solutions were further diluted to 10 μM with methanol.

DMISC derivatization solutions were prepared at a concentration of 20 mg/mL in acetone in accordance with previous studies [31,33,39]. The solution was subjected to ultrasonic treatment for 15 min to improve the dissolution of DMISC. Following this, the solution was centrifuged at 3320 × *g* for 10 min, and the resulting supernatant was utilized for further derivatization.

### 4.3. Biotransformation with 19 Human Recombinant UGTs by Enzyme Bags Method

Fission yeast strains were cultured and incubated in Edinburgh Minimal Medium (EMM) supplemented with leucine following the protocols described in our previous studies [28,41] (see Supplementary Text S1). The EMM was prepared in-house based on the published protocol [42]. After the culturing, the yeast cell density was counted under the microscope. The calculated volume of liquid medium containing 5 × 10^7^ yeast cells was used for a single enzyme bag reaction [28,41]. Following centrifugation at 3320 × *g* for 5 min, the supernatant was carefully discarded, and the cell pellets were permeabilized with 1 mL of 0.3% Triton X-100 in Tris-KCl buffer. The samples were then incubated at 30 °C with agitation at 230 rpm for one hour. After permeabilization, the cell pellets were washed three times with 1 mL of NH_4_HCO_3_ buffer (50 mM, pH 7.8) to remove the residual detergent.

Subsequently, the washed cell pellets (referred to as enzyme bags), were resuspended in 200 μL of phosphate-buffered saline (PBS) containing 50% glycerol (*v*/*v*). Finally, the enzyme bag samples were flash frozen by liquid nitrogen and stored at −80 °C. The feasibility of utilizing preserved enzyme bags was previously demonstrated [43]. All enzyme bags used in this investigation were frozen for less than three months, which is a suitable storage duration with acceptable activity loss [43]. For the investigation of glucuronidation the frozen enzyme bags were thawed on ice and washed twice with NH_4_HCO_3_ buffer to eliminate glycerol. Subsequently, the enzyme bags were suspended in 200 μL NH_4_HCO_3_ buffer (50 mM, pH 7.8) containing 1 mM UDPGA and 1 mM substrate, and were incubated for 15 h at 37 °C and 1000 rpm in the incubator. After the reaction, the samples were centrifuged at 14,100 × *g* for 5 min, and the supernatants were collected for further LC-MS/MS analysis.

### 4.4. Incubation with Human Liver Microsomes

In vitro incubations with HLMs were performed in two groups for two purposes: firstly, to generate reference samples with an incubation time of 4 h for identifying glucuronides in urine or enzyme bag samples. Secondly, to produce sufficient amounts of hydroxypropranolol glucuronides with a longer incubation time of 24 h for derivatization reaction.

For generating reference samples only containing (*R*)-form glucuronides, (*R*)-propranolol was used as the substrate for synthesizing the respective diastereomeric hydroxypropranolol glucuronides via HLM-catalyzed enzymatic reactions since enantiopure 5- and 7-OHP were not available. The reactions required NADPH regeneration system and UDPGA as cofactors to enable both phase I and phase II metabolism. A total of 5 μL of pooled human liver microsomes (5 mg/mL) was added to a 50 mM NH_4_HCO_3_ buffer containing 3 mM MgCl_2_, 1 mM (*R*)-propranolol, 1 mM UDPGA, and 1× NADPH regeneration system, which is composed of a 1× concentration of solution A containing NADP^+^ and glucose-6-phosphate, and a 1× concentration of solution B containing glucose-6-phosphate dehydrogenase. The final reaction volume was 200 μL. The reaction mixture was incubated at 37 °C with agitation at 1000 rpm for 4 h, and then centrifuged at 14,100 × *g*. The supernatant was collected for analysis. To prepare reference samples of 4-, 5-, and 7-OHPG, the NADPH regeneration system was not required, and the substrate was replaced with the respective 4-, 5-, or 7-OHP at the same concentration (1 mM). The other components of the reaction mixture remained unchanged.

To generate sufficient amounts of hydroxypropranolol glucuronides, 5 μL of pooled human liver microsomes (5 mg/mL) were incubated with cofactors and substrates for a longer time of 24 h. The rest of the reaction buffer was 50 mM NH_4_HCO_3_ buffer containing 3 mM MgCl_2_, 1 mM hydroxypropranolol, and 1 mM UDPGA. The reaction mixture was incubated at 37 °C with agitation at 1000 rpm. After incubation, 400 μL acetonitrile was added to the system, and the samples were centrifuged at 14,100 × *g* for 5 min to obtain the supernatant. Aliquots of 300 μL of the supernatant were utilized for the derivatization reaction, while the remaining portion of the supernatant was preserved as underivatized samples for comparison with the derivatized samples.

### 4.5. Urine Collection

Urine samples were collected over a period of seven days following the administration of a single dose of 40 mg racemic propranolol. During the first 24 h, continuous urine collection was performed, while for the subsequent days, only morning urine samples were collected. All urine samples were stored at −20 °C until further analysis. Before analysis, 200 μL of each urine sample were diluted with 800 μL of methanol. The diluted samples were then subjected to centrifugation at 3320× *g* for 10 min. The resulting supernatants were collected for subsequent analysis.

All urine samples were analyzed by LC-MS/MS and the abundances of analytes are listed in Supplementary Table S1. Among the collected urine specimens, the sample obtained at 17.6 h after the oral administration of propranolol was specifically chosen for comparison with the incubations with HLMs and enzyme bags. This is because 4, 5- and 7-OHPGs were found in this urine sample and their amounts were higher than in the other urine samples.

### 4.6. Derivatization with DMISC

The derivatization method used in this study was adapted from a previous publication by Solomonsen et al. [31]. Before the derivatization process, 300 μL of either a 10 μM substrate solution or the supernatant from HLM incubations were evaporated to dryness under N_2_ flow in a heating block at 65 °C. Subsequently, 100 μL of sodium carbonate buffer (0.1 M, pH 10.5) was added to the dried residues, and the tubes were vortexed for 1 min to dissolve the residue. Next, 100 μL of DMISC solution (20 mg/mL in acetone) were added. After incubation in a 60 °C water bath for 10 min the samples were cooled to room temperature and filtered through 0.45 μm filters (CHROMAFIL^®^RC-45/15 MS, Macherey-Nagel GmbH & Co., Ltd. KG Düren, Germany) for subsequent LC-MS/MS analysis.

### 4.7. LC-MS/MS Analysis

#### 4.7.1. LC-QQQ-MS/MS

The analysis was conducted using an Agilent Technologies 1290 Infinity high-performance liquid chromatograph coupled with an Agilent 6495 Triple Quadrupole mass spectrometer (Agilent Technologies, Waldbronn, Germany). Separation of the substrates and metabolites was achieved using an Agilent ZOBRAX Eclipse Plus C18 column with dimensions of 100 mm × 2.1 mm and a particle size of 1.8 μm.

The separation of 4-OHP, 5-OHP, 7-OHP, and their respective glucuronides was achieved using a flow rate of 0.4 mL/min and a column temperature of 30 °C. The mobile phase consisted of 95% water with 20 mM ammonium formate (A) and 5% methanol with 20 mM ammonium formate as starting conditions. Over a period of 25 min, the percentage of A was linearly reduced to 60%. From 25 to 26 min, the percentage of A was further decreased to 5%, and this concentration was maintained until the end of the 28-min run. A post-run time of 2.5 min at 95% of A was performed for re-equilibration.

In order to separate 4-MeOP, 5-MeOP, 7-MeOP, and their respective glucuronides, similar conditions were applied. Aberrantly, for the gradient mobile phase A was decreased from the starting conditions to 60% within 25 min, further reduced to 40% during 25 to 30 min, and to 5% from 30 to 31 min. Following this, A was maintained at 5% until the end of the run, which was 33 min in total.

Details of the transitions of analytes can be found in Table 6, with the presented peak areas provided by transitions of the highest intensity.

#### 4.7.2. LC-QTOF-MS/MS

The separation and structural confirmation of DMIS derivatives was performed using an Agilent Technologies 1290 Infinity high-performance liquid chromatograph coupled to an Agilent 6550 QTOF mass spectrometer (Agilent Technologies, Santa Clara, CA, USA). The column employed was an Agilent ZOBRAX Eclipse Plus C18 column with dimensions of 100 mm × 2.1 mm and a particle size of 1.8 μm.

The LC elution method for separating DMIS derivatives of propranolol and hydroxypropranolols was adapted from a previous study [31]. The flow rate was set at 0.35 mL/min, and the column temperature was maintained at 30 °C. The initial mobile phase composition was 98% water with 0.1% formic acid and 10 mM ammonium formate (A) and 2% acetonitrile with 0.1% formic acid, 10% water, and 10 mM ammonium formate (B). The amount of A was decreased to 85% within 7 min, to 60% within 7 to 14 min, and 5% within 14 to 15 min. Then, A maintained at 5% until the end of the run, 17 min.

The separation of DMIS derivatives of 4-, 5- and 7-OHPGs was achieved using a gradient with a flow rate of 0.2 mL/min and a column temperature of 35 °C. The initial composition of the mobile phase consisted of 80% water containing 20 mM ammonium formate (A) and 20% methanol with 20 mM ammonium formate (B). Subsequently, the proportion of component B was gradually increased to 32% over a period of 14 min, followed by a further increase to 43% between 14 to 25 min. After this, B was raised to 90% and maintained until the end of the 30-min run.

For the structural confirmation of glucuronides and DMIS derivatives, targeted MS/MS was performed. MS parameters were set using electrospray ionization in positive ion mode (ESI+) with spectra acquired over a mass range of *m/z* 100–1000; capillary voltage 3500 V; drying gas temperature 200 °C; drying gas flow 12 L/min. The specific collision energies used in each particular analysis are presented in the individual figure legends.

## 5. Conclusions

In this study, the diastereomeric glucuronic metabolites of 4-, 5-, and 7-OHP were successfully separated and identified in human urine. To further understand the specific UGT isoforms responsible for the glucuronidation of 5-OHP and 7-OHP, a comprehensive reaction phenotyping was conducted using 19 enzymes from the UGT1 and UGT2 families. The results revealed the involvement of UGT1A1, UGT1A3, UGT1A7, UGT1A8, UGT1A9, UGT1A10, UGT2A1, and UGT2A2 in the glucuronidation of 5-hydroxypropranolol. Additionally, UGT1A6 exhibited a weak glucuronidation activity towards 7-hydroxypropranolol, along with the previously mentioned eight UGT isoforms. Pre- and post-glucuronidation derivatization methods demonstrated that both aromatic and aliphatic hydroxy groups of hydroxypropranolol are able to be glucuronidated under in vitro conditions. However, hydroxynaphthyl glucuronidation is the preferred pathway under physiological conditions.

## Figures and Tables

**Table 1:** The activity of 19 human UGTs towards racemic 5- or 7-hydroxypropranolol.

UGTs	(*S*)-5-OHPG	(*R*)-5-OHPG	(*S*)-7-OHPG	(*R*)-7-OHPG
UGT1A1	+	+	+	+
UGT1A3	+	+	+	+
UGT1A4	-	-	-	-
UGT1A5	-	-	-	-
UGT1A6	-	-	+	+
UGT1A7	+ + + +	+ + + +	+ + + + +	+ + + +
UGT1A8	+ +	+	+	+
UGT1A9	+ + + +	+ + +	+ + + + +	+ + + +
UGT1A10	+	+	+	+
UGT2A1	+ + +	+ + +	+ + + +	+ +
UGT2A2	+	+	+	+
UGT2A3	-	-	-	-
UGT2B4	-	-	-	-
UGT2B7	-	-	-	-
UGT2B10	-	-	-	-
UGT2B11	-	-	-	-
UGT2B15	-	-	-	-
UGT2B17	-	-	-	-
UGT2B28	-	-	-	-

“+” for peak area < 5000; “+ +” for peak area 5000–20,000; “+ + +” for peak area 20,000–50,000; “+ + + +” for peak area 50,000–250,000; “+ + + + +” for peak area > 250,000; “-” not detected; (*S*)- or (*R*)- 5-/7-OHPG, (*S*)- or (*R*)- 5-/7-hydroxypropranolol glucuronide.

**Table 2:** The activity of 9 human UGTs toward racemic 5- or 7-methoxypropranolol.

UGTs	5-MeOPG I	5-MeOPG II	7-MeOPG I	7-MeOPG II
UGT1A1	+ +	+ +	+	+ +
UGT1A3	+ +	+ +	+ +	+ +
UGT1A6	**	**	-	-
UGT1A7	+ + +	+ + + +	+ + +	+ + + +
UGT1A8	-	-	-	-
UGT1A9	+ + + +	+ + + +	+ + + +	+ + + +
UGT1A10	+ + +	+	+ +	+
UGT2A1	+ + + +	+ + + +	+ + + +	+ + + +
UGT2A2	-	-	-	-

“+” for peak area < 100; “+ +” for peak area 100–1000; “+ + +” for peak area 1000–10,000; “+ + + +” for peak area > 10,000; “**” The substrate was not tested by respective UGT; “-” not detected; 5-MeOPG, 5-methoxypropranolol glucuronide; 7-MeOPG, 7-methoxypropranolol glucuronide.

**Table 3:** Postulated fragments, their elementary composition, theoretical mass, observed mass, and the resulting mass differences for (*R*)-propranolol-DMIS, corresponding to Figure 5A.

Postulated Fragment	Elementary Composition	Theoretical Mass (*m/z*)	Experimental Mass (*m/z*)	Mass Error (ppm)
[M+H]^+^	[C_21_H_28_N_3_O_4_S]^+^	418.1795	418.1797	0.48
[M+H–C_3_H_6_]^+^	[C_18_H_22_N_3_O_4_S]^+^	376.1326	376.1332	1.60
[M+H–C_3_H_6–_H_2_O]^+^	[C_18_H_20_N_3_O_3_S]^+^	358.1220	358.1212	–2.23
[M+H–C_10_H_8_O]^+^	[C_11_H_20_N_3_O_3_S]^+^	274.1220	274.1214	–2.19
[M+H–C_10_H_8_O–C_3_H_6_]^+^	[C_8_H_14_N_3_O_3_S]^+^	232.0750	232.0752	0.86
[M+H–C_10_H_8_O–C_3_H_6–_H_2_O]^+^	[C_8_H_12_N_3_O_2_S]^+^	214.0645	214.0642	–1.40
[DMIS-NCH_2_+H]^+^	[C_6_H_10_N_3_O_2_S]^+^	188.0488	188.0487	–0.53
[DMIS-NH_2_+H]^+^	[C_5_H_10_N_3_O_2_S]^+^	176.0488	176.0488	0.00

**Table 4:** Postulated fragments, their elementary composition, theoretical mass, observed mass, and the resulting mass differences for mono-DMIS derivative of 5-hydroxypropranolol, corresponding to Figure 5B.

Postulated Fragment	Elementary Composition	Theoretical Mass (*m/z*)	Experimental Mass (*m/z*)	Mass Error (ppm)
[M+H]^+^	[C_21_H_28_N_3_O_5_S]^+^	434.1744	434.1750	1.38
[M+H–C_3_H_6_]^+^	[C_18_H_22_N_3_O_5_S]^+^	392.1275	392.1274	−0.26
[M+H–C_3_H_9_N–H_2_O]^+^	[C_18_H_17_N_2_O_4_S]^+^	357.0907	357.0897	−2.8
[M+H–DMIS]^+^	[C_16_H_20_NO_3_]^+^	274.1438	274.1447	3.28
[M+H–DMIS–C_3_H_6_]^+^	[C_13_H_14_NO_3_]^+^	232.0968	232.0966	−0.86
[M+H–DMIS–C_3_H_9_N]^+^	[C_13_H_11_O_3_]^+^	215.0703	215.0703	0.00
[M+H–DMIS–C_10_H_8_O]^+^	[C_6_H_14_NO]^+^	116.1072	116.1072	0.00

**Table 5:** Postulated fragments, their elementary composition, theoretical mass, observed mass, and the resulting mass differences for double-DMIS-derivatized 5-hydroxypropranolol, corresponding to Figure 5C.

Postulated Fragment	Elementary Composition	Theoretical Mass (*m/z*)	Experimental Mass (*m/z*)	Mass Error (ppm)
[M+H]^+^	[C_26_H_34_N_5_O_7_S_2_]^+^	592.1894	592.1892	−0.33
[M+H–C_3_H_6_]^+^	[C_23_H_28_N_5_O_7_S_2_]^+^	550.1425	550.1429	0.73
[M+H–DMIS]^+^	[C_21_H_26_N_3_O_5_S]^+^	432.1588	432.1575	−3.00
[M+H–C_3_H_6_–DMIS]^+^	[C_18_H_20_N_3_O_5_S]^+^	390.1118	390.1118	0.00
[M+H–DMIS–C_3_H_9_N–H_2_O]^+^	[C_18_H_17_N_2_O_4_S]^+^	357.0904	357.0894	−2.80
[M+H–DMIS–C_10_H_8_O]^+^	[C_11_H_20_N_3_O_3_S]^+^	274.1220	274.1217	−1.09
[M+H–DMIS–C_10_H_8_O–C_3_H_6_]^+^	[C_8_H_14_N_3_O_3_S]^+^	232.0750	232.0752	0.86
[NH_2_-(CH_3_)_2_-DMIS]^+^	[C_8_H_16_N_3_O_2_S]^+^	218.0958	218.0952	−2.75
[M+H–2DMIS–C_3_H_9_N–H_2_O]^+^	[C_13_H_11_O_2_]^+^	199.0754	199.0749	−2.51
[DMIS-NCH_2_+H]^+^	[C_6_H_10_N_3_O_2_S]^+^	188.0488	188.0486	−1.06
[DMIS-NH_2_]^+^	[C_5_H_10_N_3_O_2_S]^+^	176.0488	176.0483	−2.84

**Table 6:** Transitions for all analytes in MRM.

Analytes	Retention Time (min)	Precursor Ions (*m/z*)	Product Ions (*m/z*)	CE (eV)	ESI
4-Methoxypropranolol	27.6	290	187	12	+
5-Methoxypropranolol	25.1		116	16	
7-Methoxypropranolol	27.6		72	44	
(*R*)-4-Hydroxypropranolol glucuronide	8.7	452	276	12	+
(S)-4-Hydroxypropranolol glucuronide	8.5		199	20	
(*R*)-5-Hydroxypropranolol glucuronide	6.4		173	24	
(S)-5-Hydroxypropranolol glucuronide	6.7		116	28	
(*R*)-7-Hydroxypropranolol glucuronide	17.3		98	40	
(S)-7-Hydroxypropranolol glucuronide	16.6		72	44	
4-Methoxypropranolol glucuronide I	13.3	466	290	25	+
4-Methoxypropranolol glucuronide II	13.6		213	30	
5-Methoxypropranolol glucuronide I	24.4		187	30	
5-Methoxypropranolol glucuronide II	26.1		116	28	
7-Methoxypropranolol glucuronide I	25.7		72	44	
7-Methoxypropranolol glucuronide II	27.0				

CE, collision energy; ESI, electrospray ionization; Drying gas temperature, 160 °C; Drying gas flow, 11 L/min; Nebulizer, 30 psi; Sheath gas heater, 375 °C; Sheath gas flow, 12 L/min; Capillary, 3000 V; Nozzle Voltage, 1500 V.

## Data Availability

The data presented in this study are available on request from the corresponding author.

## Supplementary Materials

Supporting information can be downloaded at: https://www.mdpi.com/article/10.3390/molecules28237783/s1. Figure S1: Product ion spectra (LC-QTOF-MS/MS) of (*R*)-4-hydroxypropranolol glucuronide (A) and (*R*)-7-hydroxypropranolol glucuronide (B), obtained from HLM incubations. (*R*)-4-hydroxypropranolol glucuronide, C_22_H_29_NO_9_, [M+H]^+^ theor. = 452.1915, [M+H]^+^ exp. = 452.1895, Δ*m/z* = –4.42 ppm; collision energy 30 eV. (*R*)-7-hydroxypropranolol glucuronide, C_22_H_29_NO_9_, [M+H]^+^ theor. = 452.1915, [M+H]^+^ exp. = 452.1918, Δ*m/z* = 0.66 ppm; collision energy 30 eV. The blue diamond indicated the precursor ion; Figure S2: Product ion spectra (LC-QTOF-MS/MS) of 4-methoxypropranolol glucuronide (A) and 7-methoxypropranolol glucuronide (B) obtained from UGT1A9 enzyme bag incubations. 4-methoxypropranolol glucuronide, C_23_H_31_NO_9_, [M+H]^+^ theor. = 466.2072, [M+H]^+^ exp. = 466.2061, Δ*m/z* = –2.36 ppm; collision energy 30 eV. 7-methoxypropranolol glucuronide, C_23_H_31_NO_9_, [M+H]^+^ theor. = 466.2072, [M+H]^+^ exp. = 466.2058, Δ*m/z* = –3.00 ppm, collision energy 30 eV. The blue diamond indicated the precursor ion; Figure S3: Extracted ion chromatograms obtained from MS full-scan analysis (LC-QTOF-MS) of DMIS derivatives of 4-hydroxypropranolol. (A) 4-OHP-DMIS, mono-DMIS derivative of 4-hydroxypropranolol, [M+H]^+^ *m/z* 434. (B) 4-OHP-2DMIS, bis-DMIS derivative of 4-hydroxypropranolol, [M+H]^+^ *m/z* 592; Figure S4: Product ion spectra (LC-QTOF-MS/MS) of 4-hydroxypropranolol-DMIS derivatives. (A) 4-hydroxypropranolol-DMIS, C_21_H_27_N_3_O_5_S, corresponding to the peak in Supplementary Figure S3A, [M+H]^+^ theor. = 434.1744, [M+H]^+^ exp. = 434.1750, Δ*m/z* = 1.38 ppm, collision energy 30 eV. (B) 4-hydroxypropranolol-2DMIS, C_26_H_33_N_5_O_7_S_2_, corresponding to the peak in Supplementary Figure S3B, [M+H]^+^ theor. = 592.1894, [M+H]^+^ exp. = 592.1889, Δ*m/z* = –0.84 ppm, collision energy 30 eV. DMIS, dimethylimidazole-4-sulfonyl. The blue diamond indicated the precursor ion; Figure S5: Extracted ion chromatograms obtained from MS full-scan analysis (LC-QTOF-MS) of DMIS derivatives of 7-hydroxypropranolol. (A) 7-OHP-DMIS, mono-DMIS derivative of 7-hydroxypropranolol, [M+H]^+^ *m/z* 434. (B) 7-OHP-2DMIS, bis-DMIS derivative of 7-hydroxypropranolol, [M+H]^+^ *m/z* 592; Figure S6: Product ion spectra (LC-QTOF-MS/MS) of 7-hydroxypropranolol-DMIS derivatives. (A) 7-hydroxypropranolol-DMIS, C_21_H_27_N_3_O_5_S, corresponding to the peak in Supplementary Figure S5A, [M+H]^+^ theor. = 434.1744, [M+H]^+^ exp. = 434.1750, Δ*m/z* = 1.38 ppm, collision energy 20 eV. (B) 7-hydroxypropranolol-2DMIS, C_26_H_33_N_5_O_7_S_2_, corresponding to the peak in Supplementary Figure S5B, [M+H]^+^ theor. = 592.1894, [M+H]^+^ exp. = 592.1898, Δ*m/z* = 0.68 ppm, collision energy 30 eV. DMIS, dimethylimidazole-4-sulfonyl. The blue diamond indicated the precursor ion; Figure S7: Postulated structures of the obtained fragments from LC-QTOF-MS/MS analysis of 5-hydroxypropranolol glucuronide DMIS I, corresponding to Figure 7A in main text; Figure S8: Postulated structures of the obtained fragments from LC-QTOF-MS/MS analysis of 5-hydroxypropranolol glucuronide DMIS III, corresponding to Figure 7B in main text; Figure S9: Product ion spectra from target MS/MS analysis (LC-QTOF-MS/MS) of DMIS derivatives of 7-hydroxypropranolol glucuronides obtained from HLM incubations. (A) 7-hydroxypropranolol glucuronide DMIS I, C_27_H_35_N_3_O_11_S, [M+H]^+^ theor. = 610.2075, [M+H]^+^ exp. = 610.2083, ∆*m/z* = 2.83 ppm, collision energy 30 eV. (B) 7-hydroxypropranolol glucuronide DMIS II, C_27_H_35_N_3_O_11_S, [M+H]^+^ theor. = 610.2075, [M+H]^+^ exp. = 610.2085, ∆*m/z* = 2.94 ppm, collision energy 30 eV. The blue diamond indicated the precursor ion; Figure S10: Extracted ion chromatogram ([M+H]^+^ *m/z* 610) from MS full scan (LC-QTOF-MS) of HLMs generated (*S*)-4-hydroxypropranolol glucuronides derivatized with DMISC. (*S*)-4OHPG DMIS I and II, DMIS derivative of (*S*)-4 hydroxypropranolol glucuronide; Figure S11: Product ion spectra (LC-QTOF-MS/MS) of DMIS derivatives of (*S*)-4-hydroxypropranolol glucuronides derived from HLM incubations. (A) corresponding to (*S*)-4-OHPG DMIS I in Supplementary Figure S10, C_27_H_35_N_3_O_11_S, [M+H]^+^ theor. = 610.2075, [M+H]^+^ exp. = 610.2051, Δ*m/z* = –3.93 ppm, collision energy 30 eV. (B) corresponding to (*S*)-4-OHPG DMIS II in Supplementary Figure S10, C_27_H_35_N_3_O_11_S, [M+H]^+^ theor. = 610.2075, [M+H]^+^ exp. = 610.2062, Δ*m/z* = –2.13 ppm, collision energy 30 eV. The blue diamond indicated the precursor ion; Figure S12: Extracted ion chromatogram ([M+H]^+^ *m/z* 594) from MS full-scan (LC-QTOF-MS) of HLMs generated propranolol glucuronides derivatized with DMISC. (*S*)-PG-DMIS and (*R*)-PG-DMIS, DMIS derivatives of (*S*)-propranolol glucuronide and (*R*)-propranolol glucuronide. (A) incubation of racemic propranolol, (B) incubation of (*R*)-propranolol; Figure S13: Product ion spectra (LC-QTOF-MS/MS) of DMIS derivatives of propranolol glucuronides, corresponding to (*R*)-PG-DMIS in Supplementary Figure S12B. Propranolol glucuronide-DMIS, C_27_H_35_N_3_O_10_S, [M+H]^+^ theor. = 594.2116, [M+H]^+^ exp. = 594.2120, Δ*m/z* = 0.67 ppm, collision energy 30 eV. The blue diamond indicated the precursor ion; Figure S14: Postulated structures of the obtained fragments from LC-QTOF-MS/MS analysis of propranolol glucuronide-DMIS, corresponding to Supplementary Figure S13; Figure S15: Product ion spectra (LC-QTOF-MS/MS) of the peak at 16.9 min obtained from urine sample. C_22_H_29_NO_9_, [M+H]^+^ theor. = 452.1915, [M+H]^+^ exp. = 452.1950, ∆*m/z* = 7.74 ppm, collision energy 30 eV. The blue diamond indicated the precursor ion; Figure S16: Chromatogram (LC-QQQ-MS/MS) of additional 5-hydroxypropranolol glucuronides in 24-h HLM incubations using 5-OHP as substrate. The displayed ion transition is *m/z* 452→72; Figure S17: Product ion spectra (LC-QTOF-MS/MS) of the additional 5-hydroxypropranolol glucuronides in 24-h HLM incubations. C_22_H_29_NO_9_, [M+H]^+^ theor. = 452.1915, [M+H]^+^ exp. = 452.1947, Δ*m/z* = 7.08 ppm, collision energy 40 eV. The blue diamond indicated the precursor ion; Table S1: Abundance of 4-,5- and 7-hydroxypropranolol glucuronides observed in urine samples. The presented peak areas were provided by transitions of the highest intensity as indicated in Figure 1; Text S1: Yeast Culture.
