# Supplementary material for: Glucuronidation Pathways of 5- and 7-Hydroxypropranolol: Determination of Glucuronide Structures and Enzyme Selectivity
Source: Molecules. 2023 Nov 26;28(23):7783. doi: 10.3390/molecules28237783 (PMC10707847; doi:10.3390/molecules28237783)
Supplement: Supplementary file 1 [file molecules-28-07783-s001.zip › molecules-2728917-supplementary.pdf]

Glucuronidation Pathways of 5- and 7-Hydroxypropranolol: Determination of Glucuronide Structures and Enzyme Selectivity

Fan Yang[1], Maxi Wenzel[1], Matthias Bureik[2], Maria Kristina Parr[1,*]

[1] Pharmaceutical and Medicinal Chemistry (Pharmaceutical Analyses), Institute of Pharmacy, Freie Universität Berlin, Germany
[2] School of Pharmaceutical Science and Technology, Tianjin University, China
* Correspondence: maria.parr@fu-berlin.de (M.K.P)

Supplementary Material

Supplementary Figure S1. Product ion spectra (LC-QTOF-MS/MS) of (*R*)-4-hydroxypropranolol glucuronide (**A**) and (*R*)-7-hydroxypropranolol glucuronide (**B**), obtained from HLM incubations. (*R*)-4-hydroxypropranolol glucuronide, $C_{22}H_{29}NO_9$, $[M+H]^+$ theor. = 452.1915, $[M+H]^+$ exp. = 452.1895, $\Delta m/z$ = –4.42 ppm, collision energy 30 eV; (*R*)-7-hydroxypropranolol glucuronide, $C_{22}H_{29}NO_9$, $[M+H]^+$ theor. = 452.1915, $[M+H]^+$ exp. = 452.1918, $\Delta m/z$ = 0.66 ppm, collision energy 30 eV. The blue diamond indicates the precursor ion.

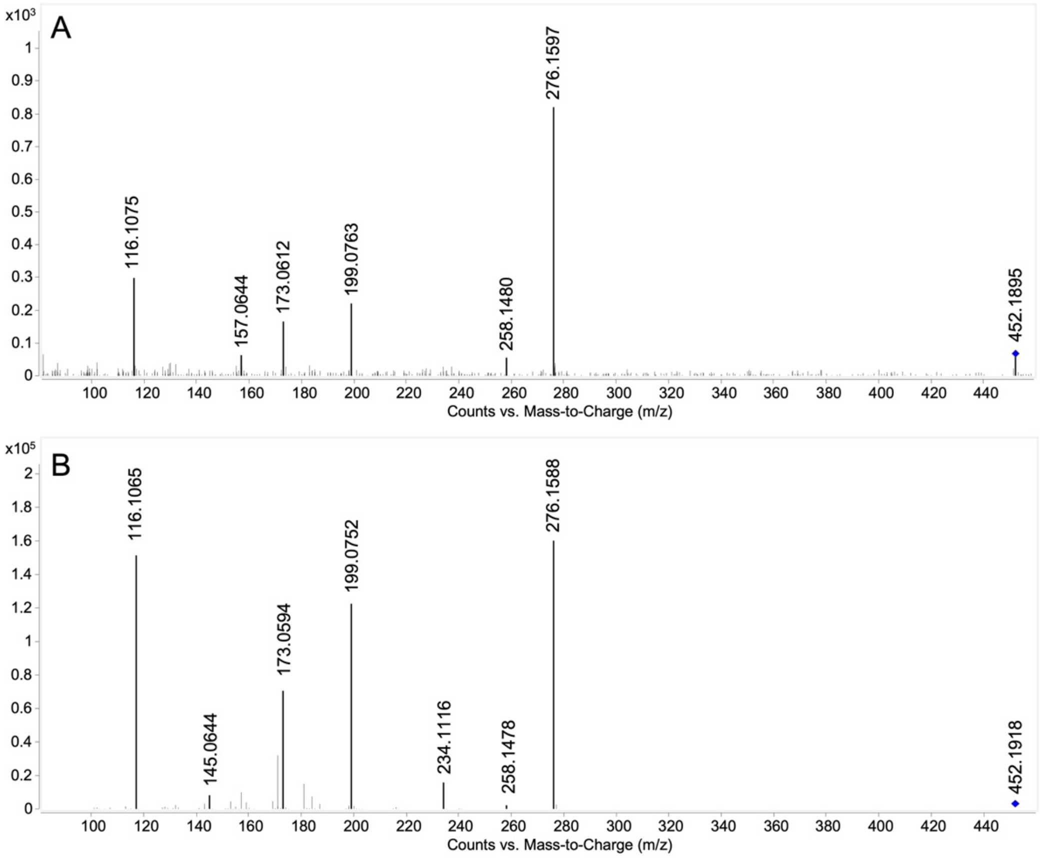

Supplementary Figure S2. Product ion spectra (LC-QTOF-MS/MS) of 4-methoxypropranolol glucuronide (**A**) and 7-methoxypropranolol glucuronide (**B**) obtained from UGT1A9 enzyme bag incubations. 4-Methoxypropranolol glucuronide, $C_{23}H_{31}NO_9$, $[M+H]^+$ theor. = 466.2072, $[M+H]^+$ exp. = 466.2061, $\Delta m/z$ = –2.36 ppm, collision energy 30 eV; 7-methoxypropranolol glucuronide, $C_{23}H_{31}NO_9$, $[M+H]^+$ theor. = 466.2072, $[M+H]^+$ exp. = 466.2058, $\Delta m/z$ = –3.00 ppm, collision energy 30 eV. The blue diamond indicates the precursor ion.

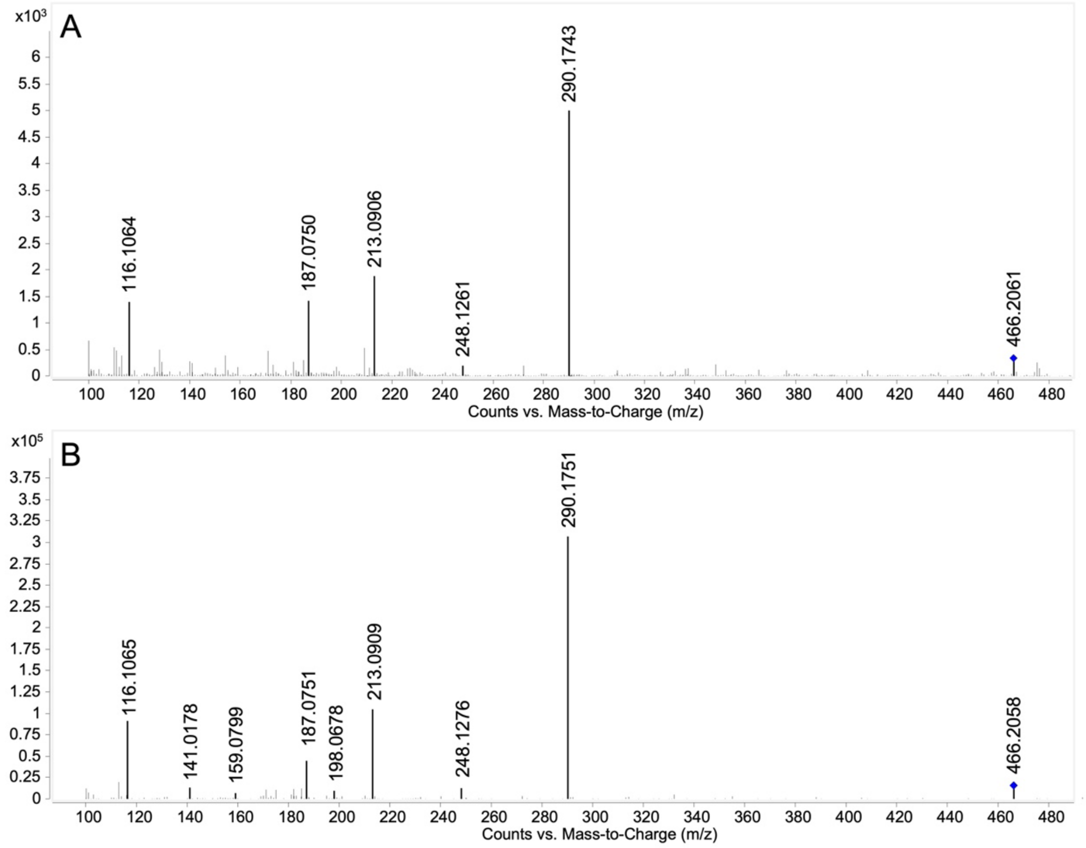

Supplementary Figure S3. Extracted ion chromatograms obtained from MS full-scan analysis (LC-QTOF-MS) of DMIS derivatives of 4-hydroxypropranolol. (**A**) 4-OHP-DMIS, mono-DMIS derivative of 4-hydroxypropranolol, $[M+H]^+$ *m/z* 434; (**B**) 4-OHP-2DMIS, bis-DMIS derivative of 4-hydroxypropranolol, $[M+H]^+$ *m/z* 592.

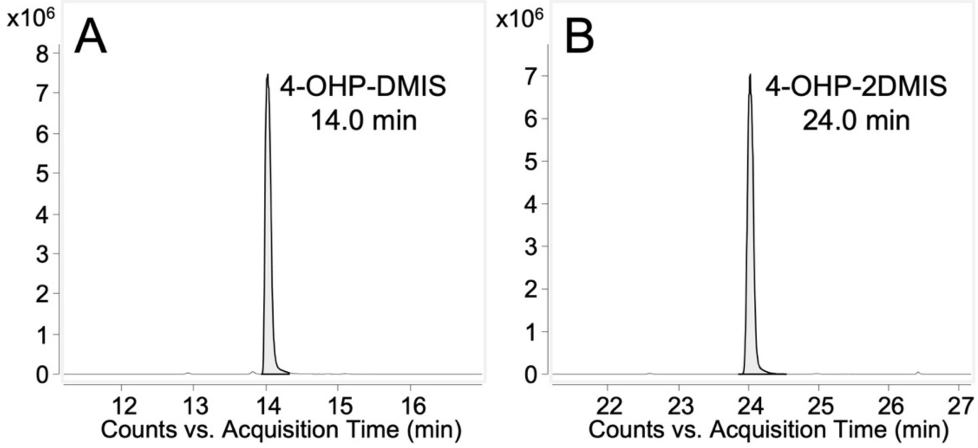

Supplementary Figure S4. Product ion spectra (LC-QTOF-MS/MS) of 4-hydroxypropranolol DMIS derivatives. (**A**) 4-Hydroxypropranolol-DMIS, $C_{21}H_{27}N_3O_5S$, corresponding to the peak in Supplementary Figure S3A, $[M+H]^+$ theor. = 434.1744, $[M+H]^+$ exp. = 434.1750, $\Delta m/z$ = 1.38 ppm, collision energy 30 eV; (**B**) 4-hydroxypropranolol-2DMIS, $C_{26}H_{33}N_5O_7S_2$, corresponding to the peak in Supplementary Figure S3B, $[M+H]^+$ theor. = 592.1894, $[M+H]^+$ exp. = 592.1889, $\Delta m/z$ = –0.84 ppm, collision energy 30 eV. DMIS, dimethylimidazole-4-sulfonyl. The blue diamond indicates the precursor ion.

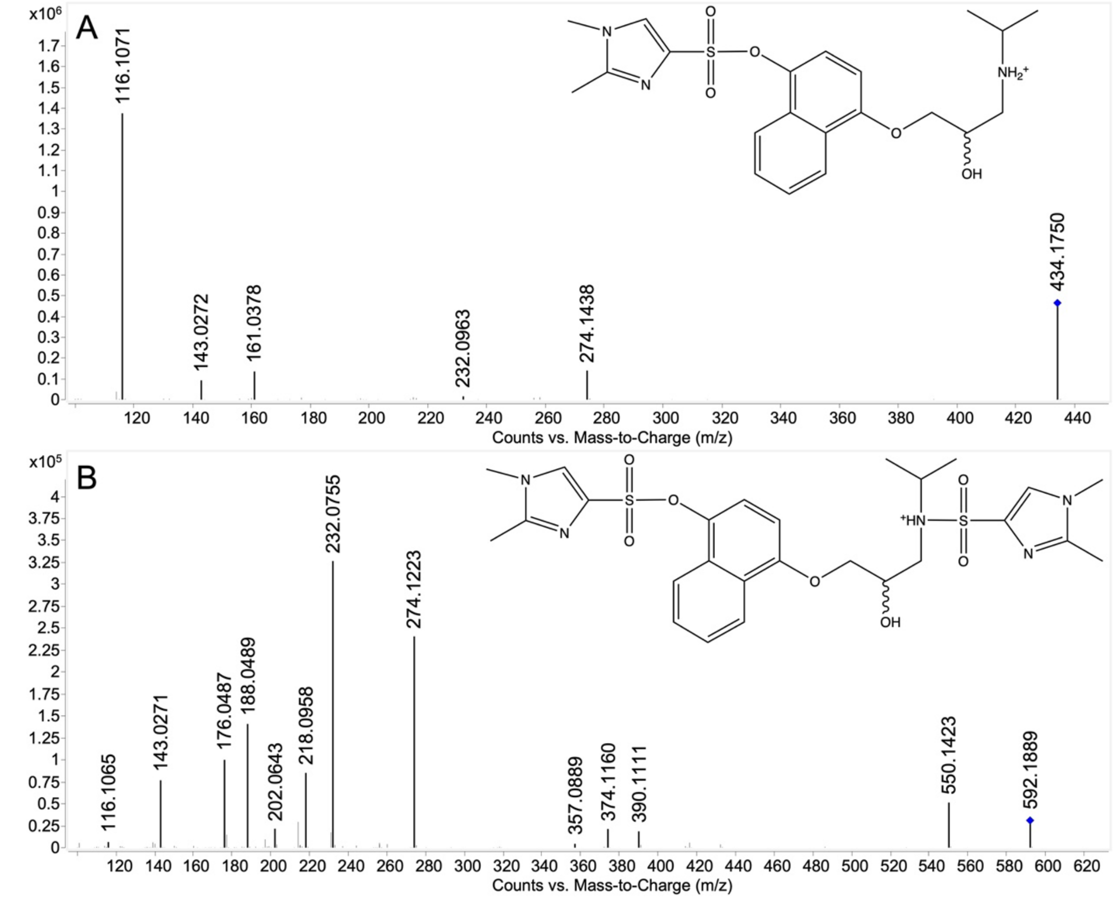

Supplementary Figure S5. Extracted ion chromatograms obtained from MS full-scan analysis (LC-QTOF-MS) of DMIS derivatives of 7-hydroxypropranolol. (**A**) 7-OHP-DMIS, mono-DMIS derivative of 7-hydroxypropranolol, $[M+H]^+$ *m/z* 434; (**B**) 7-OHP-2DMIS, bis-DMIS derivative of 7-hydroxypropranolol, $[M+H]^+$ *m/z* 592.

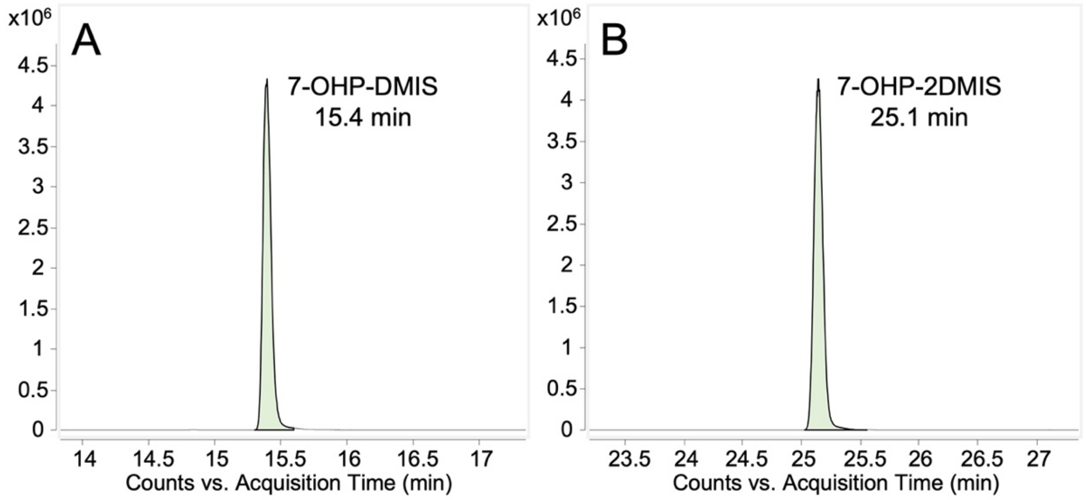

Supplementary Figure S6. Product ion spectra (LC-QTOF-MS/MS) of 7-hydroxypropranolol DMIS derivatives. (**A**) 7-Hydroxypropranolol-DMIS, $C_{21}H_{27}N_3O_5S$, corresponding to the peak in Supplementary Figure S5A, $[M+H]^+$ theor. = 434.1744, $[M+H]^+$ exp. = 434.1750, $\Delta m/z$ = 1.38 ppm, collision energy 20 eV; (**B**) 7-hydroxypropranolol-2DMIS, $C_{26}H_{33}N_5O_7S_2$, corresponding to the peak in Supplementary Figure S5B, $[M+H]^+$ theor. = 592.1894, $[M+H]^+$ exp. = 592.1898, $\Delta m/z$ = 0.68 ppm, collision energy 30 eV. DMIS, dimethylimidazole-4-sulfonyl. The blue diamond indicates the precursor ion.

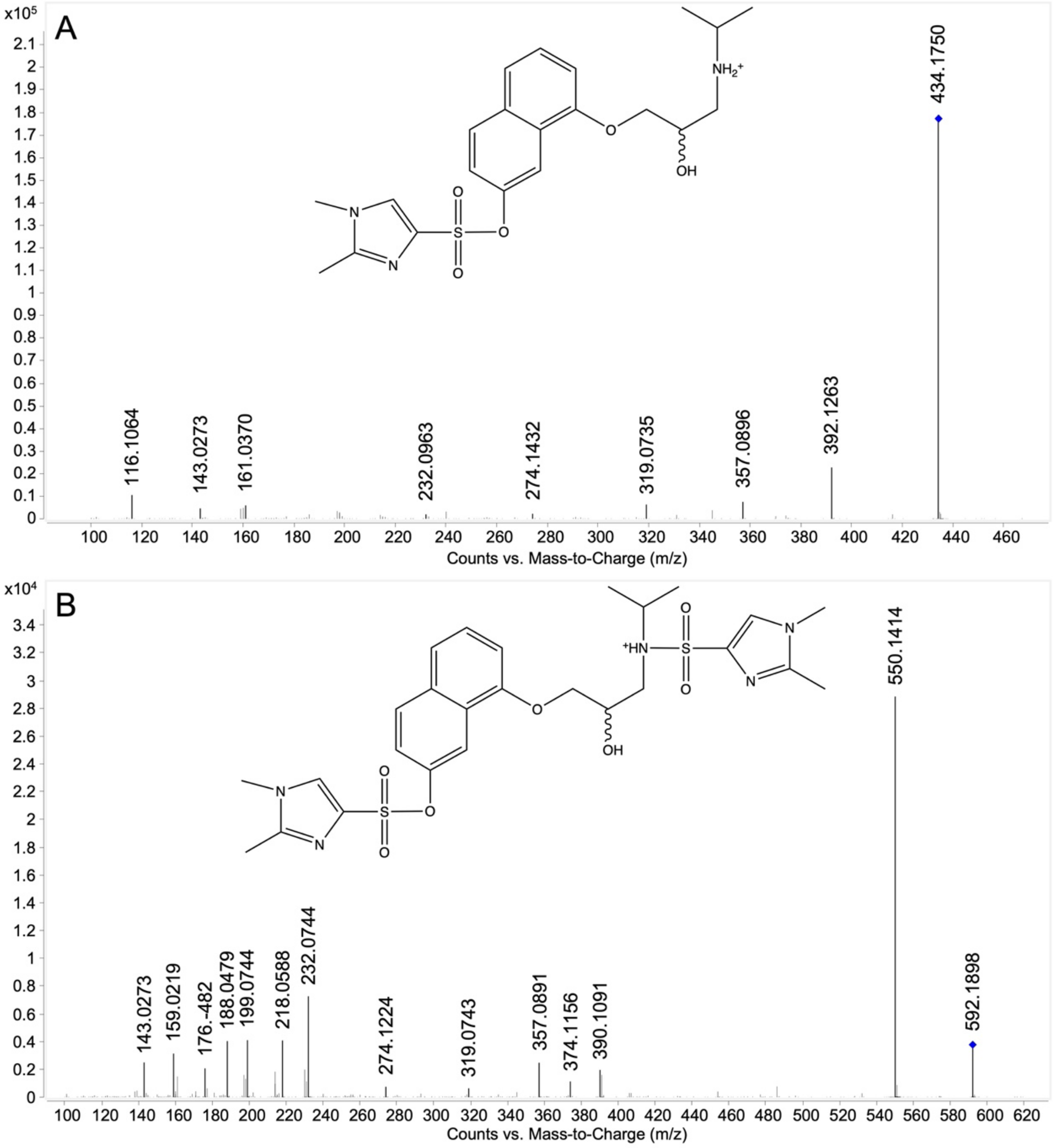

Supplementary Figure S7. Postulated structures of the obtained fragments from LC-QTOF-MS/MS analysis of 5-hydroxypropranolol glucuronide-DMIS I, corresponding to Figure 7A in main text.

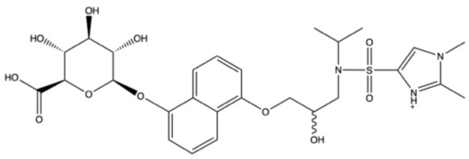

$[C_{27}H_{36}N_3O_{11}S]^+$ *m/z* 610.2050

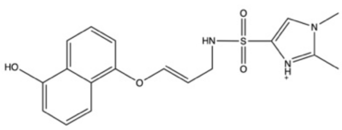

$[C_{18}H_{20}N_3O_4S]^+$ *m/z* 374.1169

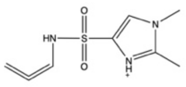

$[C_8H_{12}N_3O_2S]^+$ *m/z* 214.0645

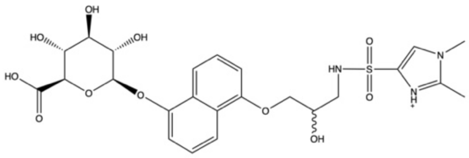

$[C_{24}H_{30}N_3O_{11}S]^+$ *m/z* 568.1596

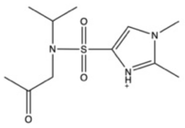

$[C_{11}H_{20}N_3O_3S]^+$ *m/z* 274.1220

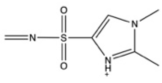

$[C_6H_{10}N_3O_2S]^+$ *m/z* 188.0488

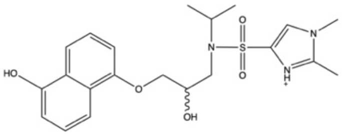

$[C_{21}H_{28}N_3O_5S]^+$ *m/z* 434.1744

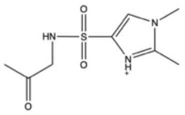

$[C_8H_{14}N_3O_3S]^+$ *m/z* 232.0750

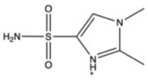

$[C_5H_{10}N_3O_2S]^+$ *m/z* 176.0488

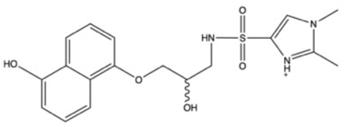

$[C_{18}H_{22}N_3O_5S]^+$ *m/z* 392.1275

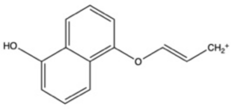

$[C_{13}H_{11}O_2]^+$ *m/z* 199.0754

Supplementary Figure S8. Postulated structures of the obtained fragments from LC-QTOF-MS/MS analysis of 5-hydroxypropranolol glucuronide-DMIS III, corresponding to Figure 7B in main text.

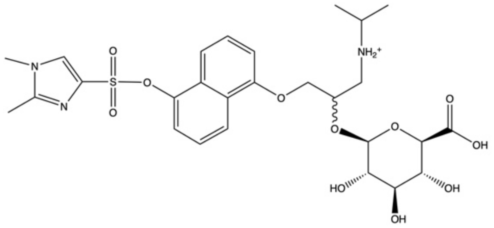

$[C_{27}H_{36}N_3O_{11}S]^+$ *m/z* 610.2050

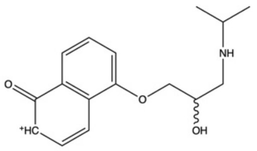

$[C_{16}H_{20}NO_3]^+$ *m/z* 274.1438

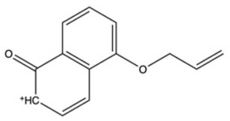

$[C_{13}H_{11}O_2]^+$ *m/z* 199.0754

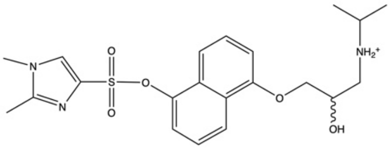

$[C_{21}H_{28}N_3O_5S]^+$ *m/z* 434.1744

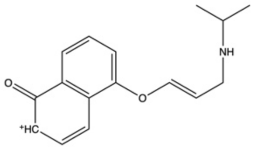

$[C_{16}H_{18}NO_2]^+$ *m/z* 256.1332

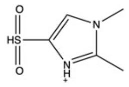

$[C_5H_9N_2O_2]^+$ *m/z* 161.0379

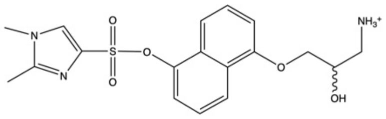

$[C_{18}H_{22}N_3O_5S]^+$ *m/z* 392.1275

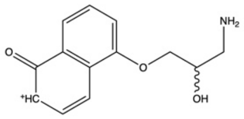

$[C_{13}H_{14}NO_3]^+$ *m/z* 232.0968

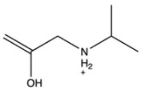

$[C_6H_{14}NO]^+$ *m/z* 116.1070

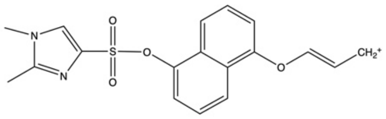

$[C_{18}H_{17}N_2O_4S]^+$ *m/z* 357.0904

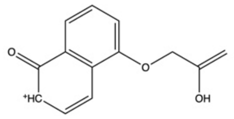

$[C_{13}H_{11}O_3]^+$ *m/z* 215.0703

Supplementary Figure S9. Product ion spectra from targeted MS/MS analysis (LC-QTOF-MS/MS) of DMIS derivatives of 7-hydroxypropranolol glucuronides obtained from HLM incubations. (**A**) 7-Hydroxypropranolol glucuronide DMIS I, $C_{27}H_{35}N_3O_{11}S$, $[M+H]^+$ theor. = 610.2075, $[M+H]^+$ exp. = 610.2083, $\Delta m/z$ = 2.83 ppm, collision energy 30 eV; (**B**) 7-hydroxypropranolol glucuronide DMIS II, $C_{27}H_{35}N_3O_{11}S$, $[M+H]^+$ theor. = 610.2075, $[M+H]^+$ exp. = 610.2085, $\Delta m/z$ = 2.94 ppm, collision energy 30 eV. The blue diamond indicates the precursor ion.

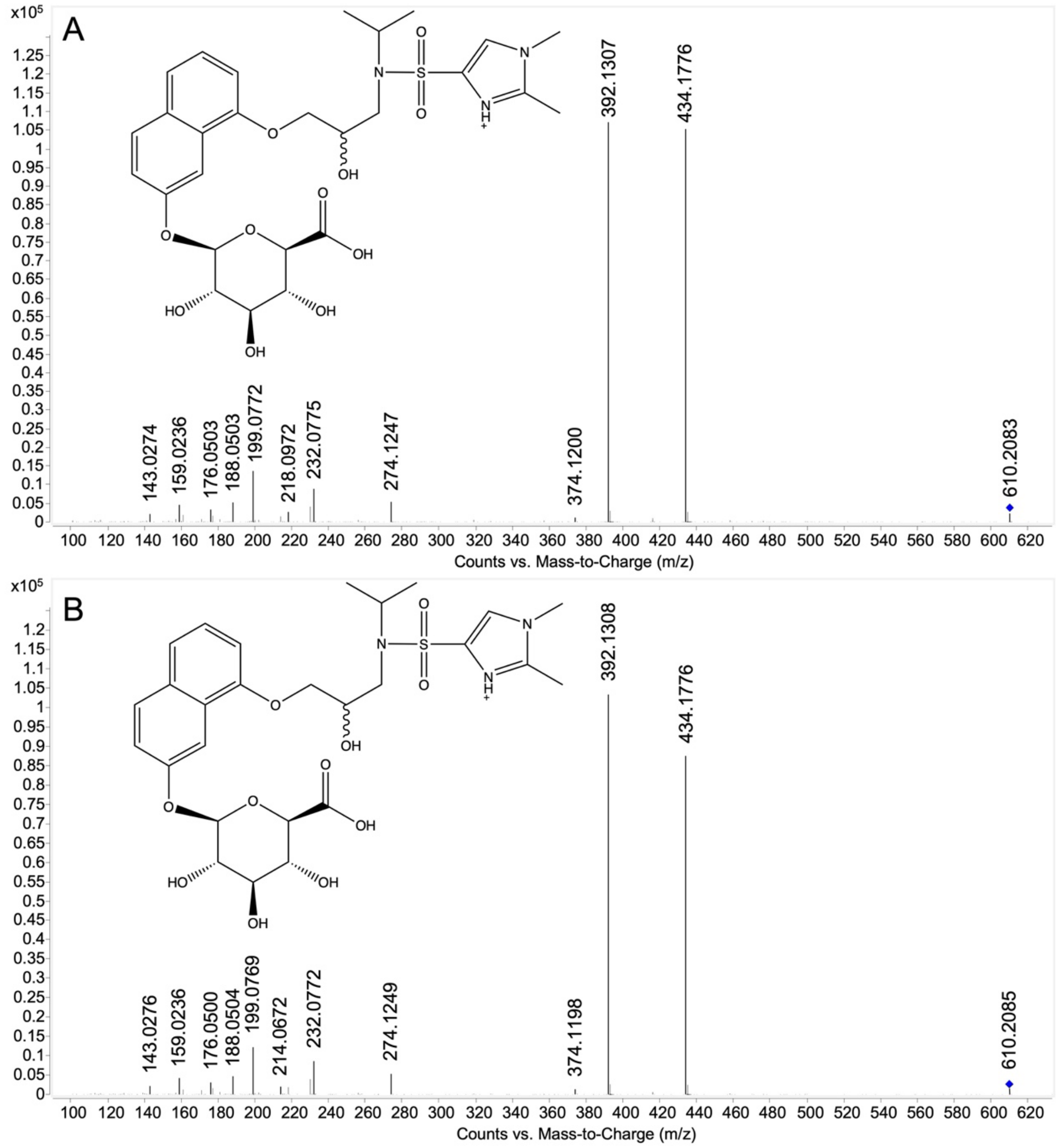

Supplementary Figure S10. Extracted ion chromatogram ([M+H]+ *m/z* 610) from MS full-scan (LC-QTOF-MS) of HLMs generated (*S*)-4-hydroxypropranolol glucuronides derivatized with DMISC. (*S*)-4OHPG DMIS I and II, DMIS derivative of (*S*)-4-hydroxypropranolol glucuronide.

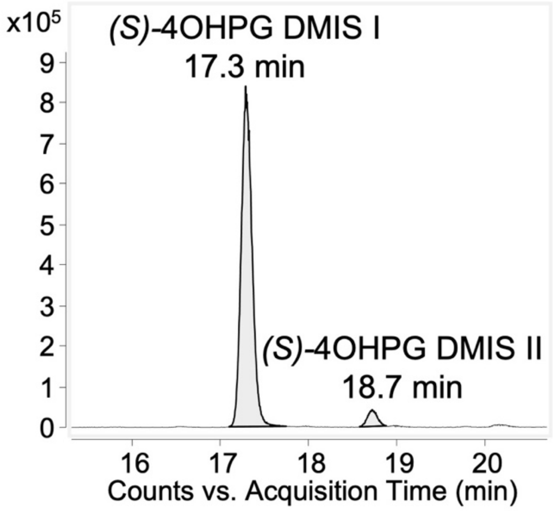

Supplementary Figure S11. Product ion spectra (LC-QTOF-MS/MS) of DMIS derivatives of (*S*)-4-hydroxypropranolol glucuronides derived from HLM incubations. (**A**) corresponding to (*S*)-4-OHPG DMIS I in Supplementary Figure S10, $C_{27}H_{35}N_3O_{11}S$, [M+H]+ theor. = 610.2075, [M+H]+ exp. = 610.2051, Δ*m/z* = –3.93 ppm, collision energy 30 eV; (**B**) corresponding to (*S*)-4-OHPG DMIS II in Supplementary Figure S10, $C_{27}H_{35}N_3O_{11}S$, [M+H]+ theor. = 610.2075, [M+H]+ exp. = 610.2062, Δ*m/z* = –2.13 ppm, collision energy 30 eV. The blue diamond indicates the precursor ion.

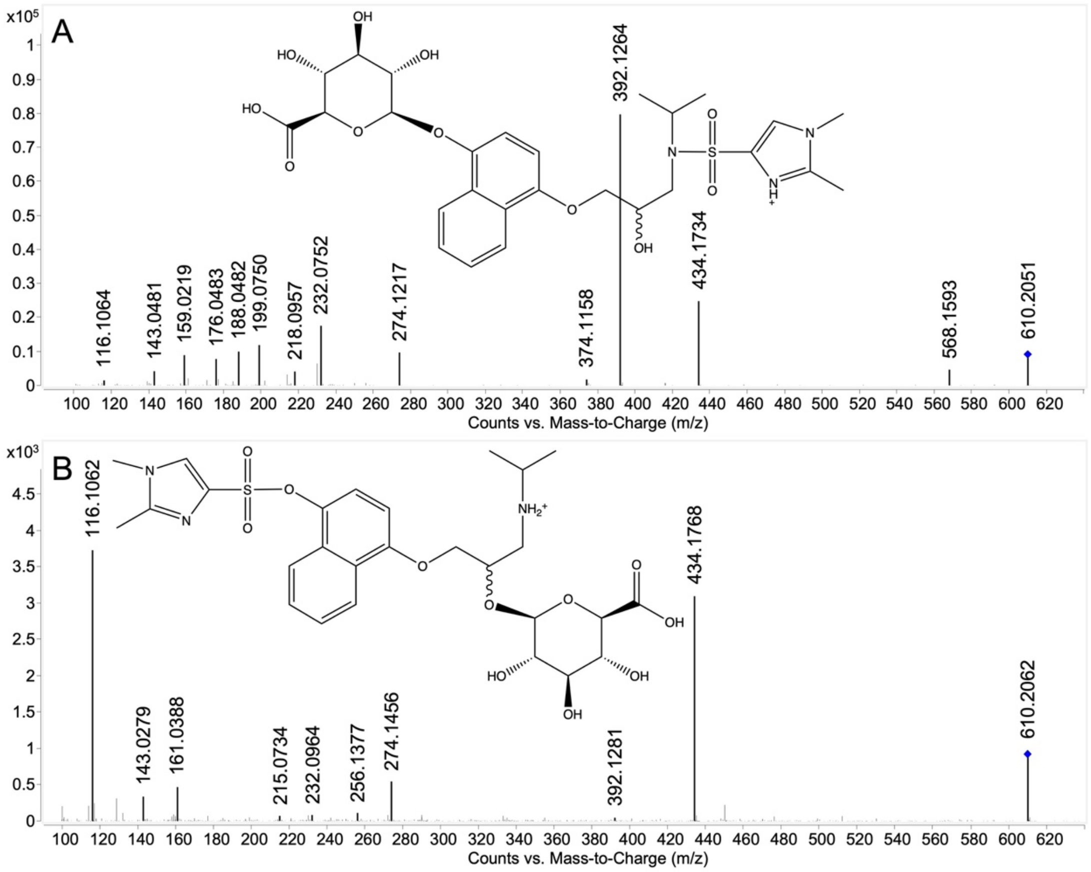

Supplementary Figure S12. Extracted ion chromatogram ($[M+H]^+$ *m/z* 594) from MS full-scan (LC-QTOF-MS) of HLMs generated propranolol glucuronides derivatized with DMISC. (*S*)-PG-DMIS and (*R*)-PG-DMIS, DMIS derivatives of (*S*)-propranolol glucuronide and (*R*)-propranolol glucuronide. (**A**) incubation of racemic propranolol; (**B**) incubation of (*R*)-propranolol.

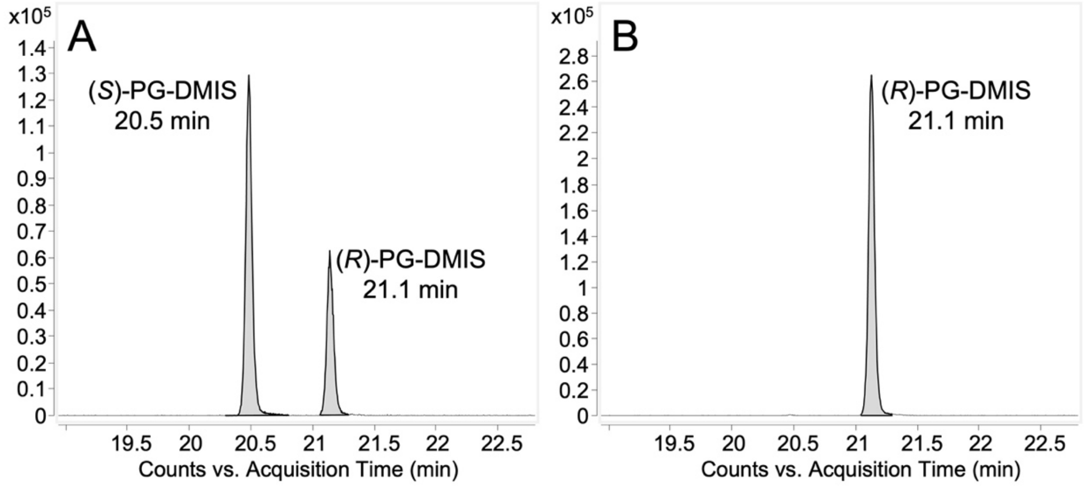

Supplementary Figure S13. Product ion spectra (LC-QTOF-MS/MS) of DMIS derivatives of propranolol glucuronides, corresponding to (*R*)-PG-DMIS in Supplementary Figure S12B. Propranolol glucuronide-DMIS, $C_{27}H_{35}N_3O_{10}S$, $[M+H]^+$ theor. = 594.2116, $[M+H]^+$ exp. = 594.2120, $\Delta m/z$ = 0.67 ppm, collision energy 30 eV. The blue diamond indicates the precursor ion.

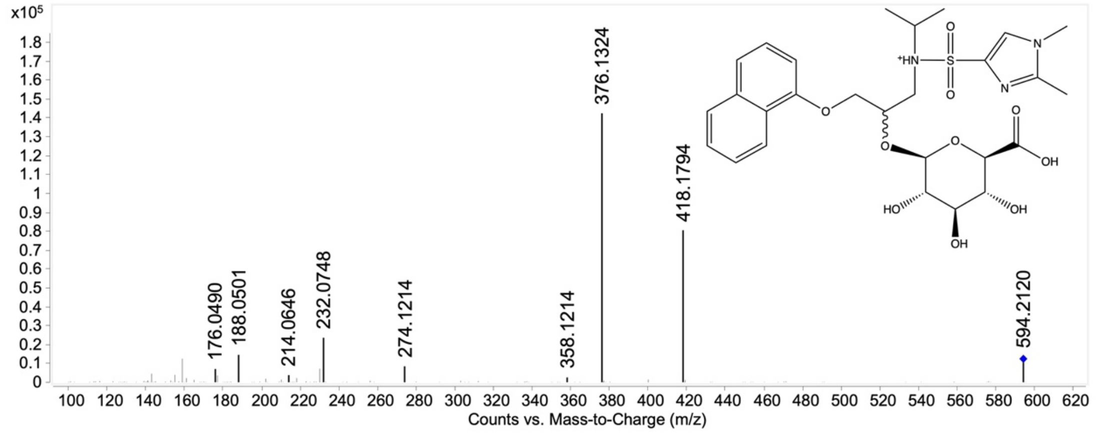

Supplementary Figure S14. Postulated structures of the obtained fragments from LC-QTOF-MS/MS analysis of propranolol glucuronide-DMIS, corresponding to Supplementary Figure S13.

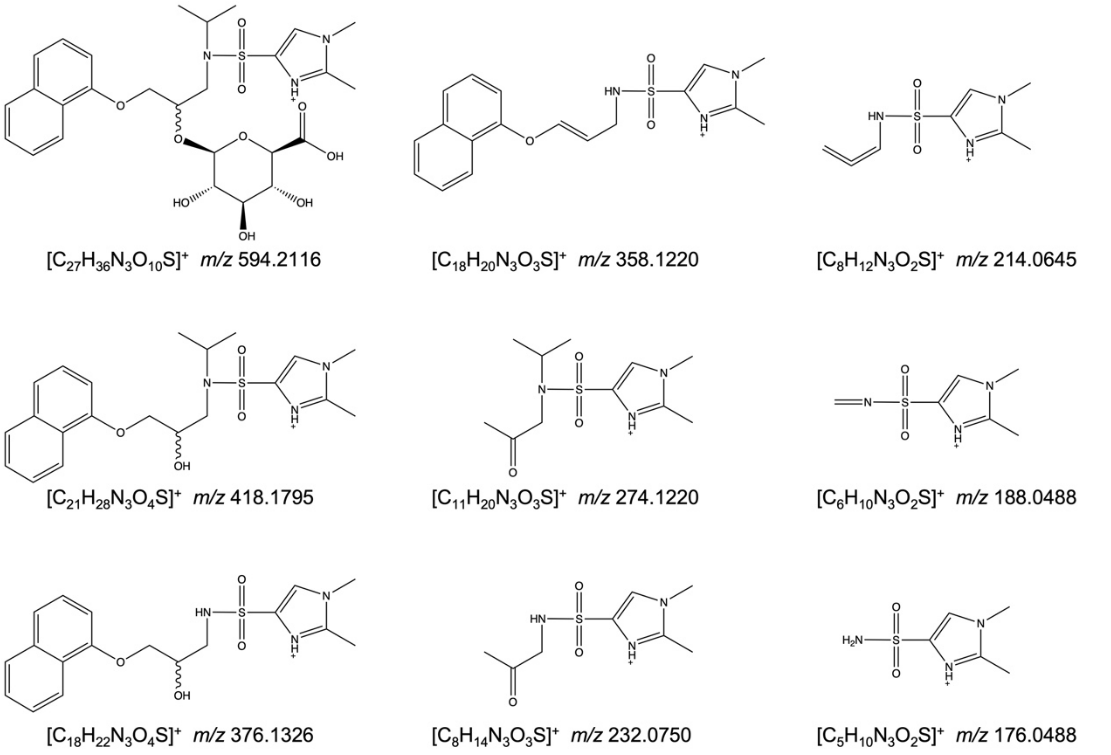

Supplementary Figure S15. Product ion spectra (LC-QTOF-MS/MS) of the peak at 16.9 min obtained from urine sample. $C_{22}H_{29}NO_9$, $[M+H]^+$ theor. = 452.1915, $[M+H]^+$ exp. = 452.1950, $\Delta m/z$ = 7.74 ppm, collision energy 30 eV. The blue diamond indicates the precursor ion.

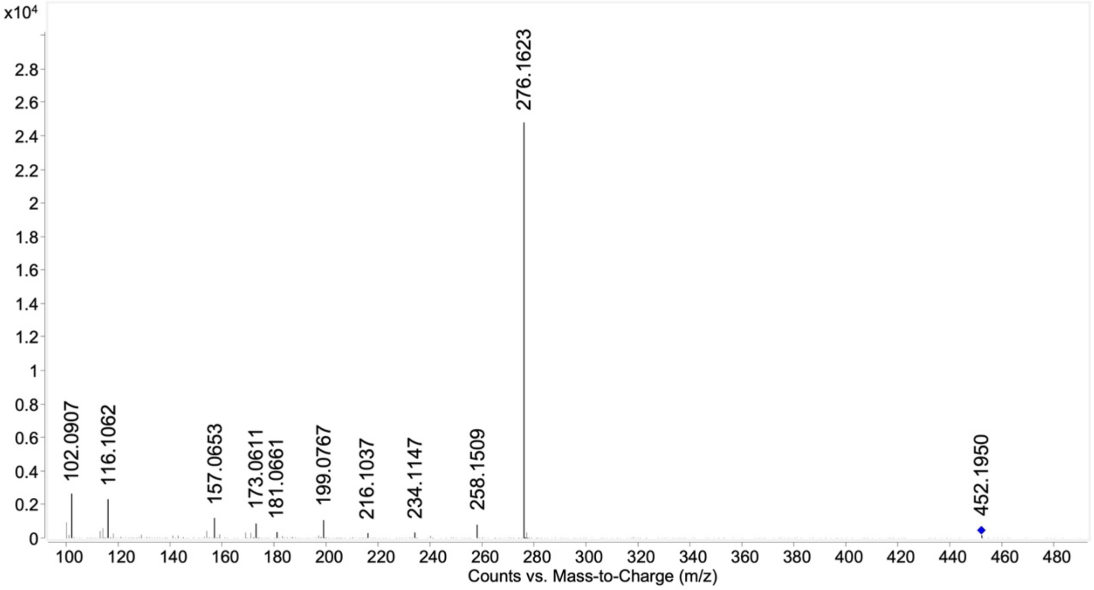

Supplementary Figure S16. Chromatogram (LC-QQQ-MS/MS) of additional 5-hydroxypropranolol glucuronides in 24-hours HLM incubations using 5-OHP as substrate. The displayed ion transition is *m/z* 452→72.

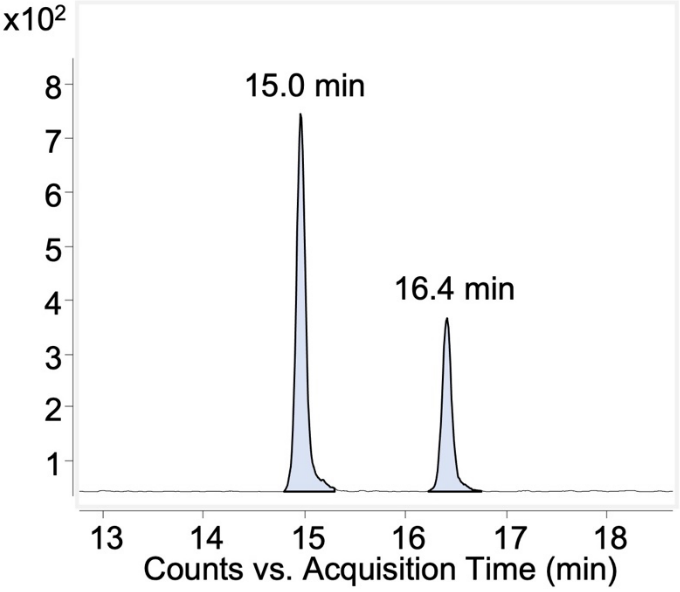

Supplementary Figure S17. Product ion spectra (LC-QTOF-MS/MS) of the additional 5-hydroxypropranolol glucuronides in 24-hours HLM incubations. $C_{22}H_{29}NO_9$, $[M+H]^+$ theor. = 452.1915, $[M+H]^+$ exp. = 452.1947, $\Delta m/z$ = 7.08 ppm, collision energy 40 eV. The blue diamond indicates the precursor ion.

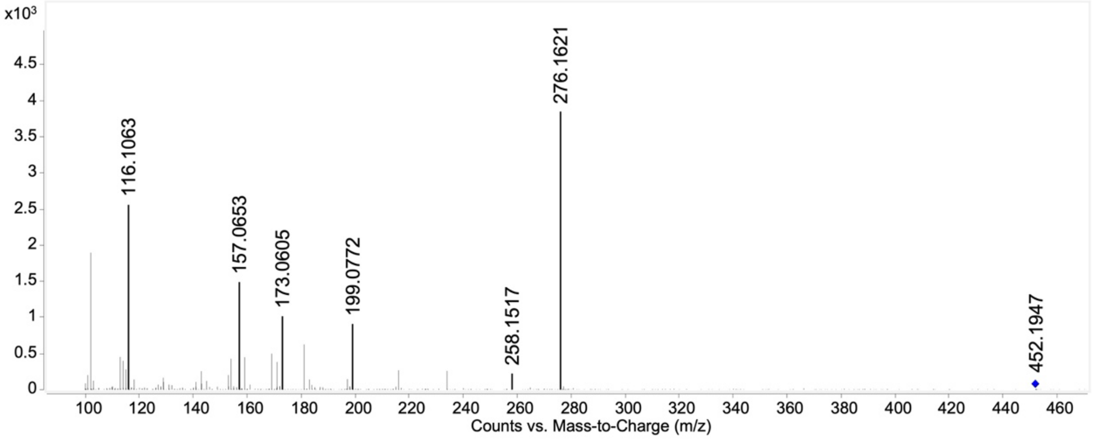

Supplementary Table S1. Abundance of 4-,5- and 7-hydroxypropranolol glucuronides observed in urine samples. The presented peak areas were provided by transitions of the highest intensity as indicated in Figure 1.

**Collected post administration (h)**	**(*S*)-4-OHPG**	**(*R*)-4-OHPG**	**(*S*)-5-OHPG**	**(*R*)-5-OHPG**	**(*S*)-7-OHPG**	**(*R*)-7-OHPG**
0.7	-	-	-	-	-	-
2.4	+ + + +	+ +	-	+	+ +	+ +
5.1	+ + + +	+ +	-	+	+ +	+ +
7.3	+ + + +	+ +	-	+	+ +	+ + +
8.2	+ + + +	+ +	-	+	+ +	+ + +
9.5	+ + + +	+ +	-	+	+ +	+ + +
13.3	+ + + + +	+ + +	-	+ +	+ +	+ + +
17.6	+ + + + +	+ + +	-	+ +	+ +	+ + + +
20.6	+ + + +	+ + +	-	+	+	+ +
23.0	+ + + +	+ +	-	+	+	+ +
38.8	+ +	+	-	-	-	+ +
61.6	+	+	-	-	-	+ +
87.5	+	+	-	-	-	+ +
98.3	-	-	-	-	-	+
167.9	-	-	-	-	-	+

“ + ” for peak area < 1,000; “ + + ” for peak area 1,000 – 10,000; “ + + + ” for peak area 10,000 – 25,000; “ + + + +” for peak area 25,000 – 40,000; “ + + + + +” for peak area > 40,000, “ - ” not detected. (*S*)- or (*R*)-4-/5-/7-OHPG, (*S*)- or (*R*)-4-/5-/7-hydroxypropranolol glucuronide.

Supplementary Text S1: Yeast culture

Initially, fission yeast strains were incubated at a temperature of 30 °C on Edinburgh Minimal Medium (EMM) solid medium supplemented with leucine for a duration of three days. Following this, the cells were transferred to 10 mL of EMM liquid medium containing leucine and agitated at 230 rpm at the same temperature for a period of 24 hours. After the initial incubation, 10 mL of the pre-culture were transferred into 100 mL of EMM liquid medium supplemented with leucine in a 200 mL flask. The flask was then placed in an incubator at 30 °C with continuous agitation at 230 rpm for a duration of 48 hours. The yeast cell density was determined using a microscope, and aliquots of 5×10^7 yeast cells were prepared for the subsequent single enzyme bags reaction.
